# Saponin-based adjuvants induce cross-presentation in dendritic cells by intracellular lipid body formation

**DOI:** 10.1038/ncomms13324

**Published:** 2016-11-07

**Authors:** Martijn H. den Brok, Christian Büll, Melissa Wassink, Annemarie M. de Graaf, Jori A. Wagenaars, Marthe Minderman, Mayank Thakur, Sebastian Amigorena, Eric O. Rijke, Carla C. Schrier, Gosse J. Adema

**Affiliations:** 1 Department of Tumor Immunology, Radboud Institute for Molecular Life Sciences, Radboud UMC, Geert Grooteplein 26, 6525 GA Nijmegen, The Netherlands; 2 Department of Anesthesiology, Pain and Palliative Medicine, Radboud UMC, Geert Grooteplein 10, 6525 GA Nijmegen, The Netherlands; 3 Institute for Laboratory Medicine, Clinical Chemistry and Pathobiochemistry, Charité Universitätsmedizin, Augustenburger Platz 1, 13353 Berlin, Germany; 4 INSERM, Institut Curie, Section Recherche, Rue d'Ulm 26, 75005 Paris, France; 5 MSD Animal Health, Wim de Korverstraat 35, 5831 AN Boxmeer, The Netherlands

## Abstract

Saponin-based adjuvants (SBAs) are being used in animal and human (cancer) vaccines, as they induce protective cellular immunity. Their adjuvant potency is a factor of inflammasome activation and enhanced antigen cross-presentation by dendritic cells (DCs), but how antigen cross-presentation is induced is not clear. Here we show that SBAs uniquely induce intracellular lipid bodies (LBs) in the CD11b+ DC subset *in vitro* and *in vivo*. Using genetic and pharmacological interference in models for vaccination and *in situ* tumour ablation, we demonstrate that LB induction is causally related to the saponin-dependent increase in cross-presentation and T-cell activation. These findings link adjuvant activity to LB formation, aid the application of SBAs as a cancer vaccine component, and will stimulate development of new adjuvants enhancing T-cell-mediated immunity.

## Abstract (web summary)

Saponin-based adjuvants are being explored as vaccine components as they induce high levels of antigen cross-presentation, but it is unknown how. Here the authors show that these adjuvants enhance cross-presentation by driving production of lipid bodies inside CD11b dendritic cells.

## Graphical abstract

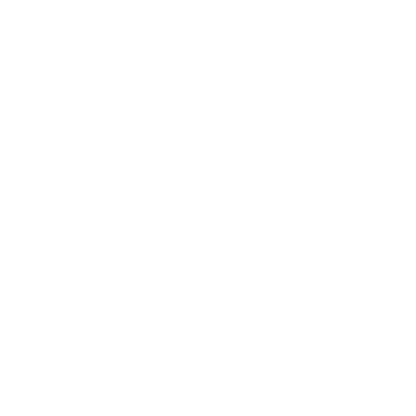

Adjuvants are diverse compounds that enhance the effectiveness of (cancer) vaccines. For human vaccines, registered adjuvants are limited and include aluminum-based and oil/water-based adjuvants. These adjuvants induce robust antibody responses, but weak cell-mediated immunity, which is crucial for application in anti-cancer vaccines. Saponin-based adjuvants (SBAs) are promising new adjuvants that enhance T-cell-mediated immunity.

Saponins are a large family of amphipathic plant glycosides, structurally sharing a lipophilic triterpene derivative. By purification of the raw plant material, fractions with distinct immunostimulatory properties and safety profiles have been identified^1^. Although most saponins have strong binding affinity for cholesterol, only some stimulate the immune system^2^. Forty nanometer cage-like particles called immune stimulatory complexes (ISCOMs) form when immunoactive saponin, cholesterol and phospholipid are brought together and dialysed^3^. The physical properties of ISCOM adjuvants contribute to its stability, and reduce the haemolytic effects associated with saponins. SBAs stimulate strong innate and adaptive cellular immunity, and elicit humoral responses of all IgG isotypes with a mixed Th1/Th2 balance^4^,^5^,^6^. By contrast, aluminum-based or oil/water-based adjuvants predominantly drive Th2 responses. Th1 responses are particularly vital for the clearance of viral infections, but also for the eradication of cancer^7^.

SBAs are now being applied in human vaccines, and several clinical trials have proven safety and efficacy^4^,^5^,^8^,^9^. Next to viral antigens (for example, H5N1), SBAs have been shown to facilitate responses to cancer antigens^10^. We previously demonstrated in our model of *in situ* tumour ablation that co-injection of SBAs induced superior anti-tumour immunity relative to other adjuvants^11^. This unique effect was accompanied by high numbers of cytotoxic T cells (CTL) specific for antigens in the ablated tumour material. Vaccination with NY-ESO-1 (ref. 12) or MAGE^13^ tumour antigens formulated in SBAs, induced strong humoral and T-cell-mediated immune responses in melanoma patients, leading to reduced relapse rates. Cebon and co-workers combined SBA vaccination with low doses of cyclophosphamide to deplete Treg cells, resulting in significantly increased T-cell responses in these patients^14^. These findings, and the improving safety profile of SBAs^15^, place SBAs at the forefront of current anti-cancer vaccine research.

Despite the growing need to understand how vaccines work, the exact immunological mechanisms of many classical immune adjuvants are not well defined. Wilson *et al*.^16^ demonstrated the involvement of the NLRP3 inflammasome and IL-18 production in ISCOM-mediated vaccine efficacy; however, this finding does not fully explain why ISCOMs stand out from other adjuvants that trigger the inflammasome (for example, Al(OH)_3_). Dendritic cells (DCs) have a critical role in the *in vivo* effectiveness of SBA-aided vaccines, and enhanced antigen cross-presentation by DCs has been reported to be particularly important for the ability of SBAs to induce cellular CD8+ T-cell immunity^17^. Two main intracellular pathways for the cross-presentation of exogenous antigens have been proposed, referred to as the ‘cytosolic' and ‘vacuolar' pathways^18^. In the cytosolic cross-presentation pathway, internalized proteins are slowly degraded in endosomal compartments by enzymatic digestion at acidic pH. By unknown mechanisms the antigens gain access to the cytosol where they are further degraded in a proteasome dependent manner. Peptides generated this way can then enter the classical MHC-I presentation route. By contrast, cross-presentation through the vacuolar pathway is proteasome independent, but sensitive to blockade of lysosomal proteolysis. Antigen processing and loading on MHC-I therefore occurs in endocytic compartments only. Cross-priming will only occur in DCs matured by interaction with pathogen-associated molecular patterns or CD4+ T helper cells^19^. Next to activation status, the sub-class of DC is also of importance. DCs can be classified as conventional DCs (cDCs), plasmacytoid DCs (pDCs) or monocyte-derived DCs^20^. cDCs are located in lymphoid and non-lymphoid tissues and can be further classified into two ontogenetically distinct subtypes: the CD8α+/CD103+ DCs and the CD11b+ DCs^21^,^22^. *In vivo* studies suggest that cross-presentation is typically performed by the CD8α+/CD103+ subset of cDCs^23^,^24^; however, under specific conditions every other subtype is capable of cross-presentation^25^. For instance, in an inflammatory environment *in vivo* CD11b+ monocyte-derived DCs efficiently cross-present OVA protein expressed by *Escherichia coli* (ref. 26). In our tumour models, SBAs trigger an unprecedented level of cross-presentation^11^, but how SBAs steer this process is unknown.

Lipid body (LB) organelles consist of a phospholipid monolayer that surrounds a core of neutral lipids, such as sterol esters or triacylglycerols. The monolayer contains numerous proteins, many with unknown function^27^. Enzymes of lipid metabolism (for example, diacylglycerol acyltransferase, DGAT), LB membrane proteins (for example, adipose differentiation-related protein, ADRP^28^), and ER proteins (for example, the p47 GTPase IGTP) have been identified in LB fractions^29^,^30^. Ralph Steinman made note of LBs in his seminal 1973 manuscript describing the dendritic cell, but their function in immune cells is only just starting to be explored^31^,^32^.

Our data now uncover an essential link between LBs and cross-presentation in the working mechanism of SBA adjuvants occurring uniquely in the CD11b+ DC subset. Genetic or pharmacological blockade of LB induction effectively abrogates the SBA-induced antigen cross-presentation, in a vaccination setting as well as an *in situ* tumour ablation model. Mechanistic studies suggest that SBAs induce LBs independent of SBA-triggered antigen translocation, and that LBs facilitate the proteasomal route of cross-presentation. These data highlight the importance of lipid body induction in CD11b+ DC for SBA vaccine activity, will aid SBA vaccine development, and the design of new adjuvants enhancing T-cell-mediated immunity.

## Results

### Saponin-based adjuvants induce cross-presentation in DCs

**Figure 1:**
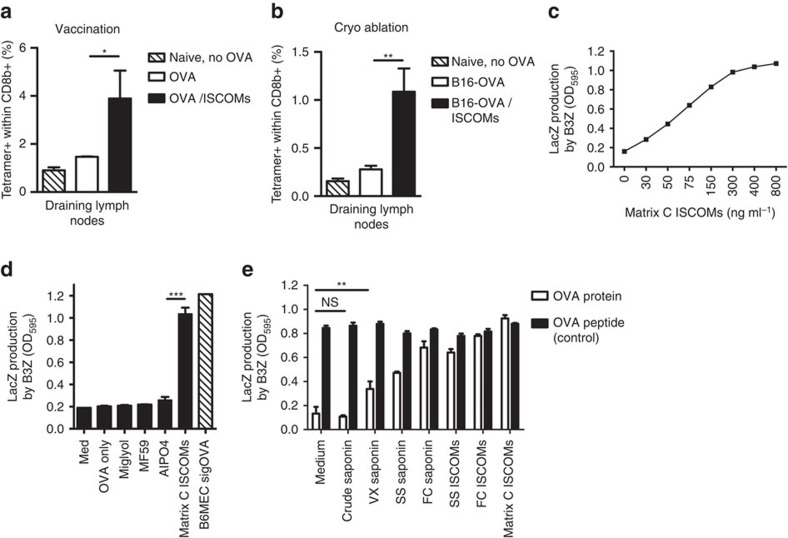
Saponin-based adjuvants induce cross-presentation in dendritic cells. (**a**,**b**) Tetramer staining of OVA specific T cells in draining lymph nodes, 10 days following s.c. injection of 20 μg OVA and/or 30 μg ISCOMs (**a**, vaccination), or 7 days following co-treatment of established B16OVA melanomas (5–8 mm diameter) with *in situ* tumour ablation and 30 μg peritumourally injected ISCOMs (**b**, Cryo-ablation). (**c**,**d**) *In vitro* B3Z cross-presentation assays demonstrating a concentration-dependent increase in cross-presentation of OVA, only when Matrix C ISCOMs was added. GM-CSF-cultured BMDCs were exposed to the indicated compounds and 80 μg ml^−1^ OVA protein for 5 h, washed and co-cultured o/n with B3Z cells, which produce β-galactosidase upon recognition of the OVA peptide in K^b^. Data in (**d**) are excerpts from larger titrations. For the non-reactive adjuvants the maximum concentration used is shown (1.5 μl ml^−1^), for the ISCOMs the concentration shown is 400 ng ml^−1^. (**e**) *In vitro* cross-presentation after 5 h exposure to 400 ng ml^−1^ of various SBAs or controls. This contains a crude saponin mixture not purified for immunoactive fractions, commercially available immunoactive saponin fractions (VX, SS and FC) and ISCOM structures made with these saponins according to an ‘open access' protocol. Passive peptide loading with OVA K^b^ peptides was used to demonstrate viability and/or equal MHC-I levels. Similar results were obtained in two to three independent experiments. Statistical analysis was done using a one or two-way ANOVA with *post hoc* Bonferroni or Tukey tests.

SBAs induce potent immune responses to a broad repertoire of antigens. SBAs and antigen can just be ad-mixed without prior complexation or formulation^4^,^5^, or simply be injected in an ablated tumour to boost antigen-specific CD8+ T-cell responses (Fig. 1a,b)^11^. In an ablation setting, the tumour debris that stays inside the body following treatment provides an effective source of (neo)antigens for processing by the immune system^33^,^34^. The induction of potent CD8+ T-cell responses is dependent on the ability of SBAs to facilitate cross-presentation of exogenous antigens in MHC-I by DCs^5^,^35^,^36^. How SBAs act to induce antigen cross-presentation is poorly understood. By testing a panel of classical non-microbial vaccine adjuvants, we first confirmed that the saponin containing ISCOM *Matrix C* induced a dramatic increase in ovalbumin (OVA) antigen cross-presentation in GM-CSF-propagated DCs (Fig. 1c; Supplementary Fig. 1a). As a read out system, we used either the co-stimulation independent B3Z reporter T-cell line^29^, or OVA-specific T cells from OT-I transgenic mice. None of the other non-microbial vaccine adjuvants was able to induce cross-presentation of OVA in DCs (Fig. 1d). The low amount of OVA used did not by itself lead to detectable cross-presentation in DCs in our assays (Fig. 1d), and MHC-II-restricted antigen presentation of OVA to OT-II cells was not altered by SBA administration (Supplementary Fig. 1b).

Only very distinct saponin fractions show immunological activity and qualify as vaccine adjuvant. So far, most SBA cross-presentation studies have been performed using proprietary made ISCOM structures^17^,^35^,^37^,^38^,^39^. Therefore, we tested whether different immune activating saponin fractions exhibited the same capacity to induce cross-presentation. All three commercially available fractions tested, and the corresponding ISCOMs made from two of these fractions according to an open-access protocol (Supplementary Fig. 2 and ref. 40) led to efficient increases in cross-presentation (Fig. 1e). In contrast, a saponin mixture not enriched for immuno-active fractions (crude saponin) did not induce cross-presentation, also at higher concentrations (Fig. 1e). Importantly, SBA treatment did not lead to reduced cell viability or MHC-I levels on the surface of the APCs, as presentation of pulsed peptide was intact in all occasions (Fig. 1e; Supplementary Fig. 3). Finally, we confirmed that within the ISCOMs the saponins are the cross-presentation inducing moiety, and not the ISCOM-stabilizing component cholesterol (Supplementary Fig. 4).

Cross-presentation induction in DCs thus is a feature shared by immunoactive saponins, but not by other saponins.

### SBA-induced cross-presentation is co-stimulation independent

**Figure 2:**
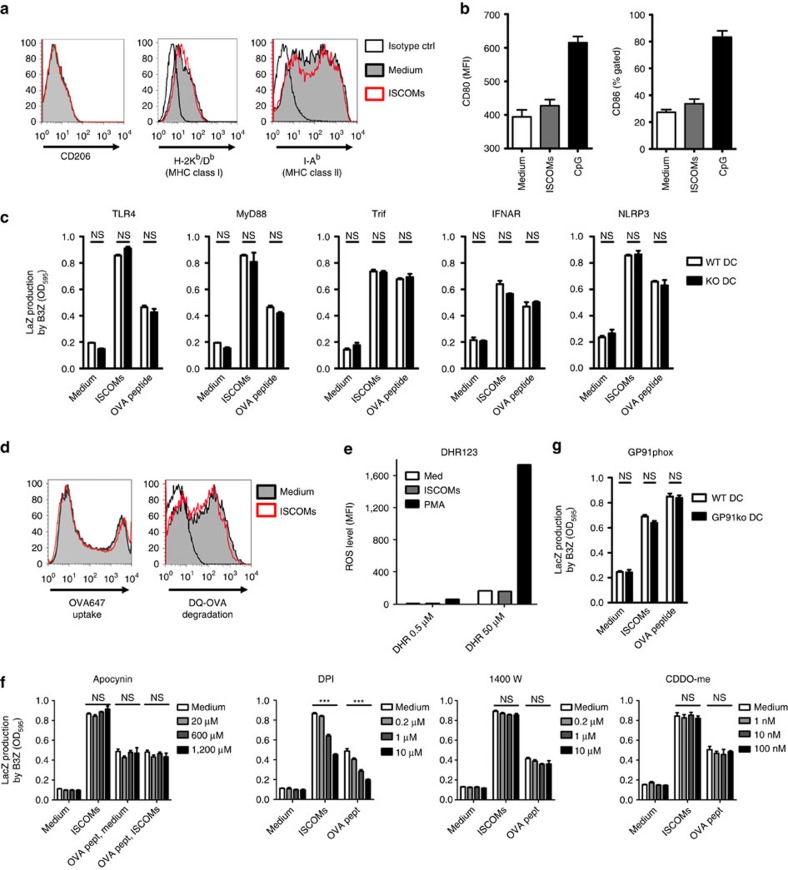
SBA-induced cross-presentation is independent of co-stimulation or ROS. (**a**) FACS analysis of surface markers CD206 (mannose receptor), MHC-I, and MHC-II. GM-CSF BMDCs were exposed for 5 h to ISCOMs or medium, and processed for FACS staining. Open black lines represent the corresponding isotype controls. Filled grey lines show the medium-treated cells, while the open red lines are the ISCOM treated samples. (**b**) FACS analysis of DC maturation markers CD80 and CD86 after 18 h stimulation with ISCOMs or 1 μg ml^−1^ CpG. (**c**) Bone-marrow of indicated knockout mice was used to generate GM-CSF BMDCs. Cells were exposed for 5 h to medium or ISCOMs, washed and co-cultured o/n with B3Z cells. External peptide pulsing (5 pg ml^−1^, 30 min) was used to control for viability and/or MHC-I levels. (**d**) Antigen uptake and processing as analysed by FACS. BMDCs were given 0.25 mg ml^−1^ OVA coupled to the fluorophore Alexa647, or 1 μg ml^−1^ DQ-OVA, during the 5 h exposure time to medium or ISCOMs. DQ-OVA will start to fluoresce once degraded. (**e**) Total cellular reactive oxygen species (ROS) were measured using the DHR123 probe. GM-CSF cultured BMDCs were plated in serum-free medium and pretreated for 10 min with 0.5 or 50 μM dihydrorhodamine 123. Next, ISCOMs or 1 μg ml^−1^ PMA was added and cells were incubated for 45 min before analysis by FACS. (**f**) *In vitro* cross-presentation in the presence of NADPH oxidase inhibitors. Indicated compounds and concentrations were added during the 5 h exposure period to ISCOMs. (**g**) *In vitro* cross-presentation using Gp91phox*−/−* bonemarrow DCs. Data in (**e**) represent single values in titration, whereas the other data show means from triplicates with s.e.m. Data are representative figures from two to three individual experiments. Statistical analyses were performed using two way ANOVA with *post hoc* Bonferroni tests.

The increases in cross-presentation could not be explained by differential surface expression of the mannose receptor (CD206, involved in uptake of OVA), or MHC-I/II (Fig. 2a), and consistent with literature, SBAs also did not change CD80 or CD86 levels on the *in vitro* cultured DCs (Fig. 2b)^17^. Together with the fact that B3Z cells react co-stimulation independent, this indicated that maturation status of the DCs is not causing the observed increases in cross-presentation. In line with this, BMDCs from *Tlr4−/−*, *Myd88−/−* or *Trif−/−* mice all had an intact ability to cross-present OVA following ISCOMs treatment (Fig. 2c). Moreover, also the type I IFN receptor or the inflammasome component NLRP3 did not affect SBA-aided cross-presentation (Fig. 2c). Antigen uptake or overall antigen processing by GM-CSF DCs, as measured by the internalization of fluorescent OVA or breakdown of DQ-OVA, was not altered following SBA treatment (Fig. 2d).

We conclude that the increases in cross-presentation induced by SBAs in DCs are antigen uptake or co-stimulation independent.

### Endosomal ROS is not involved in cross-presentation by SBAs

As previous work has implicated a role for reactive oxygen species (ROS) produced by the phagosomal NADPH-oxidase (NOX) complex in cross-presentation^41^, we set out to determine cellular ROS levels following SBA exposure. Employing two probes for detection of total ROS levels in our DCs (DHR123 and H_2_DCFDA), we found that SBA treatment for 5 h (similar to the exposure time in the cross-presentation assays) did not increase ROS levels, while the control PMA did (Fig. 2e). Although ceasing ROS production by co-treatment of the cells with commonly used concentrations of the NADPH oxidase inhibitor DPI initially seemed to abrogate the SBA-induced cross-presentation, the peptide control revealed lowered MHC-I expression due to decreased cell viability or down regulation (Fig. 2f). Three alternative NADPH oxidase inhibitors and various ROS scavengers did however not interfere with SBA-induced cross-presentation (Fig. 2f; Supplementary Fig. 5). Finally, SBAs could normally induce cross-presentation in DCs deficient in the ROS-inducing NOX2 complex component *Cybb* (GP91phox) (Fig. 2g).

Altogether these data do not support a significant role for ROS in the induction of cross-presentation by SBAs.

### SBAs induce lipid bodies in parallel with cross-presentation

**Figure 3:**
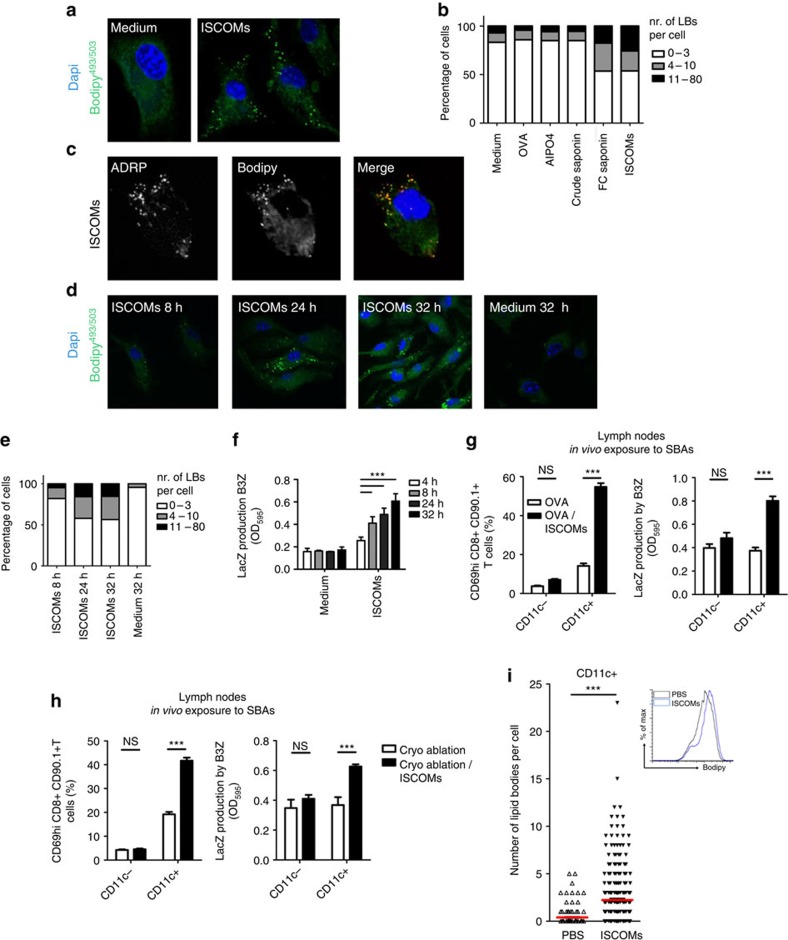
SBAs induce *in vitro* and *in vivo* lipid bodies in correlation with cross-presentation. (**a**–**c**) GM-CSF-cultured BMDCs following 5 h exposure to the indicated adjuvants were fixed and stained with the LB probe Bodipy^493/503^. Nuclei were counterstained with DAPI, and cells were analysed by confocal microscopy. (**a**) CLSM image showing SBA-induced LBs. (**b**) Quantification of lipid bodies following exposure to OVA alone, AlPO4, non-immunoactive saponin, FC saponin, or ISCOMs. Numbers of lipid bodies per cell were obtained from 90–150 cells found in 15 randomly taken CLSM images, and divided over three strata. (**c**) LB staining with counterstaining for ADRP (**d**–**f**) 32 h time-course showing concomitant increases in LB numbers (**d**,**e**) and cross-presentation (**f**). (**g**,**h**) *In vitro* cross-presentation assay on isolated lymph node CD11c+ DCs using OT-I cells (left) or B3Z cells (right) as a readout. (**g**) Three hundred microgram endotoxin-free OVA protein was injected s.c. on the femur of WT C57Bl/6 mice, with or without 30 μg ISCOMs. Twelve hours later, draining lymph nodes were harvested from which CD11c+ cells were isolated using MACS beads. Next, these cells were co-cultured with the reporter cells. CD69 levels on CD8+CD90.1+ OT-I cells were measured after 1 day, while B3Z cells were analysed after 2 days. (**h**) Established B16OVA melanomas (5–8 mm diameter) were injected i.t. with a booster shot of OVA (20 μg), after which they were treated with *in situ* tumour ablation and 30 μg peritumourally injected ISCOMs. 12 h later draining lymph nodes were harvested from which CD11c+ cells were isolated using MACS beads. Next, these cells were co-cultured with the reporter cells like in **g**. (**i**) Quantification of LBs per cell using CLSM (left) or FACS (right), on *ex vivo* CD11c+ cells as treated and isolated under (**g**). All data were reproduced in two to three independent experiments. Statistical analyses were performed using two-way ANOVA with *post hoc* Bonferroni tests, or Student's *t*-test for **i**.

Bougneres *et al*.^31^ previously demonstrated a relationship between cross-presentation in DCs and the lipid body-residing molecule IGTP. This prompted us to examine the presence of LBs in DCs exposed to various non-microbial adjuvants. To this end, we stained cells with the neutral lipid dye Bodipy^493/503^. Strikingly, SBAs (FC saponin, ISCOMs) appeared to increase the number and the brightness of cytosolic LBs as determined by confocal microscopy (CLSM), always in perfect correlation with cross-presentation levels (Figs 1d and 3a,b). Co-staining for the LB protein ADRP confirmed that the detected lipid aggregates were LBs (Fig. 3c). Other adjuvants like AlPO4, non-immunogenic crude saponin or OVA alone could not elevate the number of LBs (Fig. 3b). A time course demonstrated that the numbers of LBs per cell increased linearly over the first 32 h, which again strongly correlated with cross-presentation levels (Fig. 3d–f). Control experiments confirmed that the saponins, but not the ISCOM particle stabilizing component cholesterol represent the active moiety inducing LBs, and that mice deficient for *Tlr4*, *Nlrp3* or *Ifnar* had a normal ability to generate LBs following SBAs (Supplementary Fig. 6).

Together, these results suggest that GM-CSF DCs *in vitro* possess the striking ability to induce LBs following treatment with SBAs, which is tightly correlated with the ability to cross-present soluble antigen.

### LB formation and cross-presentation in DCs *in vivo*

To determine whether *in vivo* vaccination with SBAs also resulted in LB formation in cross-presenting DCs, we co-injected mice with ISCOMs and OVA. Twelve hours post-injection draining lymph nodes (LNs) were isolated and subjected to CD11c+ magnetic bead sorting to isolate all DCs (Fig. 3g). Similarly, DCs were isolated from B16OVA tumour bearing mice after cryo-ablation treatment with SBA co-injection (Fig. 3h). Assaying the isolated CD11c+ DC using two cross-presentation read-outs demonstrated that ISCOMs efficiently induced cross-presentation in DCs, but not the CD11c negative fraction. Evaluation of the LB content by CSLM (Fig. 3i, left) and FACS (Fig. 3i, right) showed that also after *in vivo* SBA vaccination the number of LBs increased.

### SBA sensitivity is a unique feature of monocytic CD11b+ DCs

**Figure 4:**
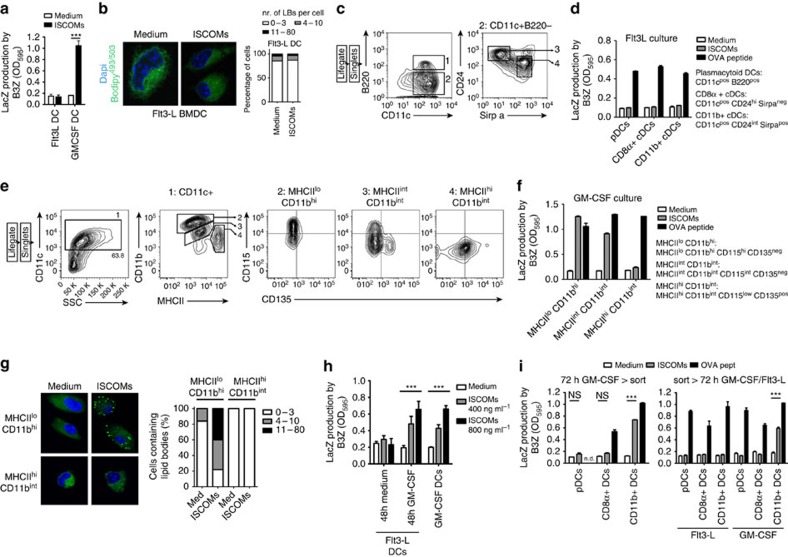
Responsiveness to SBAs *in vitro* is unique to CD11b+ DCs with monocytic origin. (**a**) *In vitro* cross-presentation assay using GM-CSF- or Flt3-L-propagated DCs. (**b**) Confocal images with LB quantifications from Flt3-L-generated BMDCs exposed for 5 h to medium or ISCOMs. (**c**) Gating strategy of Flt3-L DCs. Sorted populations are gate 1 (pDCs), gate 3 (CD8α+ DCs) and gate 4 (CD11b+ DCs). (**d**) *In vitro* cross-presentation assay using the populations as sorted under **c**. (**e**) Gating strategy of GM-CSF DCs. Sorted populations are gate 2 (MHCII^lo^ CD11b^hi^), gate 3 (MHCII^int^ CD11b^int^) and gate 4 (MHCII^hi^ CD11b^int^). (**f**) *In vitro* cross-presentation assay using the populations as sorted under **e**. (**g**) CLSM image and LB quantification of populations as sorted under **e**, after 5 h exposure to ISCOMs or medium. (**h**) *In vitro* cross-presentation assay on Flt3-L- or GM-CSF-cultured bonemarrow DCs. In the 10 day Flt3-L cultures, medium was supplemented for the last 48 h with 20 ng ml^−1^ GM-CSF or medium. (**i**) *In vitro* cross-presentation assays on Flt3-L-cultured DCs, receiving GM-CSF for 72 h after the end of culture. In the left panel the entire day 10 cultures were supplemented with GM-CSF for another 72 h, after which the resulting populations were sorted (Supplementary Fig. 8). In the right panel the day 10 cultures were sorted, after which the individual populations were treated with fresh Flt3-L or GM-CSF. All data were reproduced in two to three independent experiments. Statistical analyses were performed using two-way ANOVA with *post hoc* Bonferroni tests.

DCs can be differentiated *in vitro* from BM precursors using GM-CSF or Flt3-L. Remarkably, we found that only DCs generated with the growth factor GM-CSF, and not DCs cultured with Flt3-L, were able to cross-present antigen after SBA exposure (Fig. 4a). Increasing exposure time or dose could not change the observed cell specificity (Supplementary Fig. 7). Importantly, Flt3-L DCs readily activated B3Z cells after external pulsing with OVA K^b^ peptide (Supplementary Fig. 7b). Moreover, in these cells CD206 and MHC-I expression, and ovalbumin uptake were not influenced by the SBAs (Supplementary Fig. 7c). In line with the inability to cross-present antigen upon SBA exposure, the Flt3-L-generated DCs were incapable of inducing LBs (Fig. 4b), even at longer exposure times.

The BM cultures with Flt3-L or GM-CSF both give rise to a complex set of DC subpopulations each arising from predetermined precursors present in the starting population^42^,^43^,^44^. The observation that only 45% of the *in vitro* generated GM-CSF DCs contained LBs prompted us to purify the different DC subsets using flowcytometry, and to test their response to SBAs. First, Flt3-L DCs were sorted based on Naik *et al*.^43^ (Fig. 4c). In the three resulting DC subpopulations no population capable of cross-presenting OVA upon SBA exposure could be detected (Fig. 4d). GM-CSF DCs were sorted based on Helft *et al*.^42^, with modifications (Fig. 4e). Strikingly, only the CD135 (Flt3) negative DCs (MHCII^lo^CD11b^hi^CD115^hi^CD135^neg^ and MHCII^int^CD11b^int^CD115^int^CD135^neg^ subpopulations) could efficiently cross-present OVA in response to SBAs, whereas the CD135 positive DCs (MHCII^hi^CD11b^int^CD115^low^CD135^pos^ subset) could not (Fig. 4f). Since the MHCII^int^CD11b^int^CD115^int^CD135^neg^ cells are considered multipotent intermediates^42^, we focused on the two differentiated DC populations. Again, SBA-mediated induction of lipid bodies only occurred in the DC subset cross-presenting antigens due to SBAs: at least 80% of the MHCII^lo^CD11b^hi^CD115^hi^CD135^neg^ DCs now contained LBs after 5 h (Fig. 4g). These SBA responsive DCs were previously shown to be derived from monocytic precursors^42^. As apparently the monocytic precursors in the Flt3-L culture did not yield SBA responsive DCs, the Flt3-L culture was supplemented with GM-CSF for the last two days. Strikingly, the Flt3-L DCs now acquired full ability to cross-present antigens in response to SBAs (Fig. 4h). Sorting experiments confirmed that CD11b+ (Sirpα+) DCs, but not the CD8α+ (CD24+) DCs, were responsible for SBA-induced cross-presentation (Fig. 4i; Supplementary Fig. 8). Similarly, analysis of pDCs, and CD8α+ or CD11b+ cDCs from Flt3-L cultures that were first sorted and then individually exposed to GM-CSF confirmed that only the CD11b+ DCs gained the capacity to respond to SBAs (Fig. 4i). Stainings for the monocytic marker CD115 showed increased presence of CD115+ cells among the SBA-responsive CD11b+ DCs following GM-CSF exposure (Supplementary Fig. 8).

Collectively, these results define GM-CSF-sensitized CD11b+ DCs, presumably from monocytic origin, to be the only cells responsive to SBAs.

### Only monocytic CD11b+ DCs respond to SBAs *in vivo*

**Figure 5:**
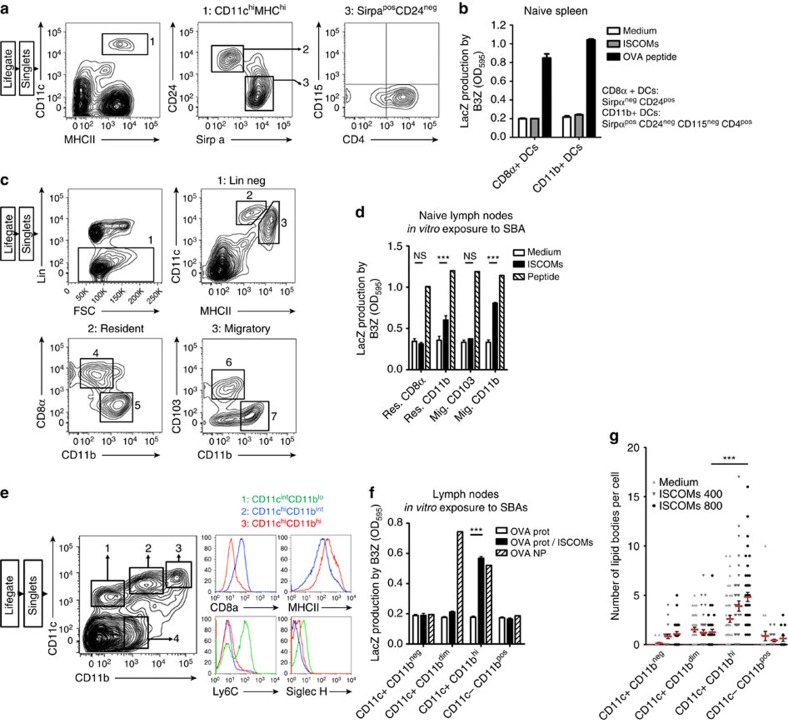
Lymph node CD11b+ DCs uniquely possess the ability to respond to SBAs* in vivo*. (**a**) Gating strategy for naive splenocytes. Sorted populations are gate 2 (CD8α+ DCs) and gate 3 (CD11b+ DCs). (**b**) *In vitro* cross-presentation assay using the populations as sorted under **a**. (**c**) Gating strategy for naïve lymph nodes. Lin- was defined as B220- and Ly6G-. Sorted populations are gate 4 (LN resident CD8α+ DCs), gate 5 (LN resident CD11b+ DCs), gate 6 (LN migratory CD103 DCs), and gate 7 (LN migratory CD11b+ DCs). (**d**) *In vitro* cross-presentation assay using the populations as sorted under **c**. (**e**–**g**) Mice were injected with Flt3-L excreting B16 melanomas to expand endogenous DC pools. After 12 days, pooled lymph nodes were harvested and subjected to FACS sorting. (**e**) Gating strategy, in which sorted populations are gates 1 to 4. Right panels show additional stainings on populations 1 to 3. (**f**) *In vitro* cross-presentation assay using populations as sorted under **e**. Sorted cells were exposed for 5 h to the indicated compounds (ISCOMs 800 ng ml^−1^, OVA 300 μg ml^−1^, PLGA nano-particles corresponding to 30 μg ml^−1^ OVA). (**g**) LB quantification in populations as sorted under **e**. All data were reproduced in two independent experiments. Statistical analyses were performed using two-way ANOVA with *post hoc* Bonferroni tests.

Next, we determined whether the selective SBA responsiveness of *in vitro* cultured DCs also exists *in vivo*. CD8α+ and CD11b+ DCs sorted from naive spleens based on the markers CD24, Sirp-α, CD115 and CD4 (Fig. 5a)^36^, could not cross-present OVA upon SBA exposure but efficiently presented externally loaded control peptides (Fig. 5b). This is not unexpected as no CD115 staining was observed on these CD11b+ DCs, indicating the absence of cells from monocytic origin, shown to be responsive to SBA *in vitro* (Fig. 5a). Therefore, resident and migratory CD8α+, CD103+ or CD11b+ DCs were sorted from naïve LNs (Fig. 5c)^21^. Subsequent exposure to SBAs demonstrated that LN resident, but especially the LN migratory CD11b+ DCs, were capable of cross-presenting OVA (Fig. 5d). The ability to respond to SBAs coincided with the presence of cells expressing the monocytic markers CD14 and Ly6C within the resident, but especially the migratory CD11b+ DCs (Supplementary Fig. 9). To evaluate a situation with a high monocytic influx, we analysed mice bearing Flt3-L excreting tumours to provoke expansion of endogenous DCs, including pDCs, in an activated environment^45^,^46^. From the tumour-draining LNs we isolated the pDC fraction, a fraction containing all CD8α+ LN-resident DCs, and the CD11b^hi^ DC fraction, and exposed them *in vitro* to SBAs (Fig. 5e). Clearly, only the CD11b^hi^ DC fraction was able to cross-present OVA upon SBA exposure (Fig. 5f). As expected, these DCs were also the only cells that contained LBs after SBA treatment (Fig. 5g). Additional FACS analysis of MHC-II levels showed that these cells were highly activated, making it difficult to discriminate migratory from resident CD11b+ cells within this gate (Fig. 5e). As an additional control, we showed that the CD8α+ DC containing fraction was able to cross-present OVA incorporated in nanoparticles, as shown previously (Fig. 5f). Finally, we sorted cells from mice treated by *in situ* tumour ablation with co-injection of the TLR9 ligand CpG-ODN. This procedure induces the influx of fresh (inflammatory) monocytes into the LNs, but not pDCs like with the Flt3-L excreting tumours. Similar to our former findings, only the CD11b^hi^ DCs could react to SBAs (Supplementary Fig. 10).

Thus, also *in vivo* monocytic CD11b+ cells uniquely harbor the ability to cross-present soluble antigens and to induce LBs upon exposure to SBAs.

### SBA-induced lipid bodies are critical for cross-presentation

**Figure 6:**
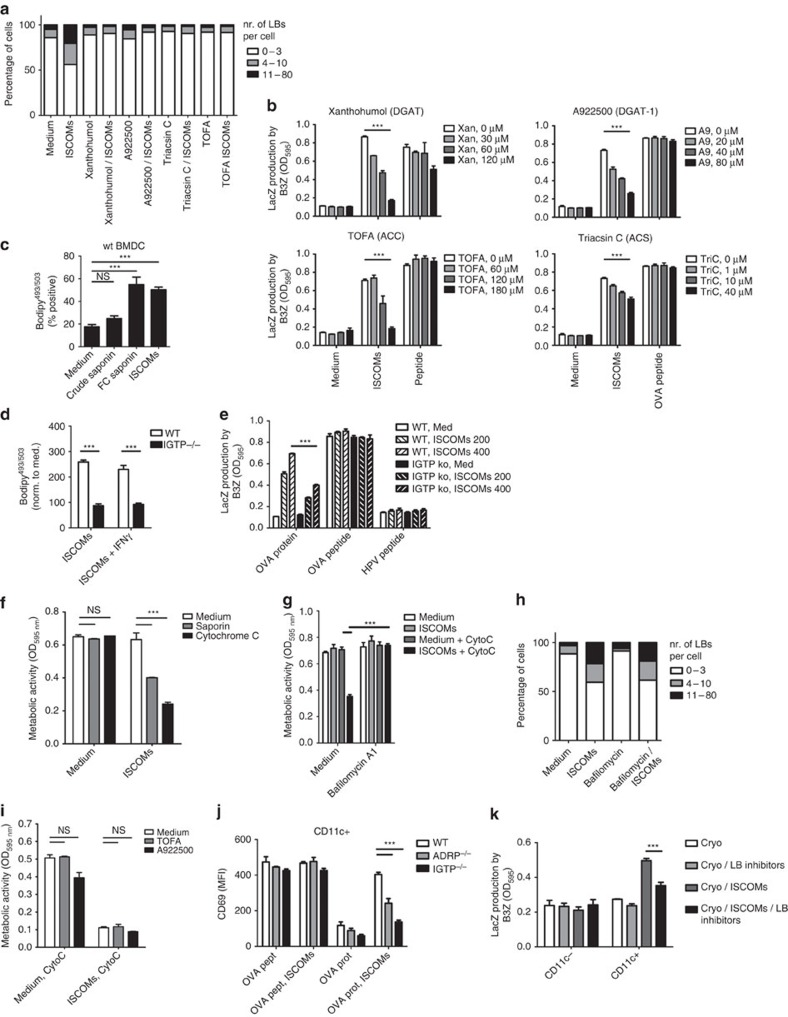
SBA-induced cross-presentation is dependent on LB formation. (**a**) LB quantification as measured by CLSM in cells treated for 5 h with the indicated LB inhibitors: (Xanthohumol: 85 μM, A922500: 40 μM, Triacsin C: 10 μM, TOFA: 120 μM). (**b**) *In vitro* cross-presentation assays in the presence of the indicated LB inhibitors (inhibited enzymes in brackets). (**c**,**d**) Flowcytometry-based analysis of LB content. Wild-type (**c**), or *Igtp+/+* and *Igtp−/−* (**d**) BMDCs were exposed to 800 ng ml^−1^ non-immunoactive saponin, FC saponin, ISCOMs or 250 ng ml^−1^ IFNγ for 5 h, washed, and stained with Bodipy^493-503^. (**e**) *In vitro* cross-presentation assay using IGTP knockout DCs. (**f**) Metabolic activity/viability (MTT) assay used to demonstrate cytosolic translocation of indicated toxins. (**g**) MTT assay showing endosomal acidification is needed for cytosolic translocation. Bafilomycin A1, (at 100 nM) is an inhibitor of endosomal v-ATPase, and thus endosomal acidification. (**h**) Quantification of LB induction in the presence of ISCOMs and Bafilomycin A1. (**i**) MTT assay showing that inhibition of LB induction does not abrogate cytosolic translocation. During the exposure to ISCOMs/Cyt C, the LB inhibitors TOFA (120 μM) and A922500 (60 μM) were added. (**j**) *In vitro* OT-I cross-presentation assay on isolated LN DCs from OVA protein or peptide-vaccinated wild-type, *Igtp−/−*, or *Adrp−/−* mice. (**k**) B16-OVA tumour bearing mice were treated with cryo-ablation, directly followed by peritumoural injection of 30 μg ISCOMs. A mixture of xanthohumol (500 μg) and A922500 (150 μg) was injected peritumourally, four hrs before ablation and directly after the ablation. 12 h later, draining LN CD11c+ cells were isolated that entered the cross-presentations assays. LacZ content in B3Z cells was analysed after 2 days. All data represent means with s.e.m., except for **d** where data represent mean MFI values normalized to medium values. Similar data were obtained in two to three independent experiments. Statistical analyses were done using ANOVA with *post hoc* Bonferroni tests.

To investigate whether a causal relationship exists between LBs and cross-presentation, we searched for ways to cease LB formation. Blocking formation of triacylglycerol, fueling the LBs, by inhibiting the rate-limiting enzymes acyl-CoA, diacylglycerol acyltransferase (DGAT) 1 and 2 or by inhibiting acyl-CoA synthetase (ACS) or acetyl-CoA carboxylase (ACC), which are further upstream in lipid synthesis, all abrogated LB formation (Fig. 6a)^31^,^47^,^48^. Importantly, inhibiting LB formation also ended the SBA dependent capacity of DCs to cross-present antigen (Fig. 6b). Viability of the cells and MHC-I levels were not affected as confirmed by external peptide loading (Fig. 6b). To further strengthen these findings, and to exclude off-target effects of pharmacological inhibition we made use of bone-marrow DCs derived from IGTP knockout mice, having a reduced capacity to generate functional LBs^31^. To screen for LB induction in a higher number of cells, we established a FACS-based protocol (Fig. 6c). As expected, *Igtp−/−* DCs had significantly less LBs compared with wild-type mice (Fig. 6d). Although Bougneres *et al*. showed that IFNγ increases expression of IGTP, and likely amplifies the molecular effects of a deficiency in IGTP^31^, the SBA-induced difference was not significantly more apparent when cells were co-treated with IFNγ. Consistent with the diminished LB levels, cross-presentation levels were reduced in the *Igtp−/−* cells, confirming the vital role for LBs in SBA-induced cross-presentation (Fig. 6e). This effect was not related to altered MHC-I or CD206 expression by DCs, or OVA processing (Supplementary Fig. 11). To validate the essential role of LBs in the observed processes, we analysed *Adrp−/−* mice also reported to have reduced functional LBs^31^,^49^. We confirmed the lower LB contents, and subsequently also found reduced SBA-induced cross-presentation (Supplementary Fig. 12).

Collectively, these results show that LB formation is essential for effective SBA-induced cross-presentation *in vitro*.

### Endosomal acidification and cytosolic antigen translocation

Enhanced endosomal antigen translocation to the cytosol has recently been proposed as a possible mechanism for SBA-induced cross-presentation^35^. Still, the position of LBs relative to cytosolic translocation, and the contribution of each process to SBA-aided cross-presentation is unclear. To assay endosomal translocation, SBA-treated DCs received the mitochondrial protein cytochrome c. Extra-mitochondrial localization of cytochrome c induces apoptosis, so translocation of endosomal cytochrome c to the cytosol will lead to reduced metabolic activity in a MTT assay. This assay, and similar experiments using the plant toxin saporin that inactivates ribosomes when cytosolic^50^, confirmed that increased cytosolic translocation took place in the presence of SBAs (Fig. 6f). Blockade of endosomal acidification showed that SBA-induced cytosolic translocation and cross-presentation were both dependent on low endosomal pH (Fig. 6g; Supplementary Fig. 13a), while treatment with the proteasome inhibitor epoxomicin demonstrated that cross-presentation also critically depended on the proteasome (Supplementary Fig. 13b). Strikingly, blockade of endosomal acidification (and thus endosomal escape) did not lower the numbers of SBA-induced LBs per cell (Fig. 6h). Conversely, pharmacological interference with LB induction also did not abrogate endosomal translocation (Fig. 6i).

These data show that SBA-triggered antigen translocation, but not SBA-induced LB formation, is critically preceded by endosomal acidification, and imply a role for SBA-induced LBs in facilitating the proteasomal route of cross-presentation.

### LBs are vital for SBA-induced cross-presentation *in vivo*

To determine if cross-presentation *in vivo*, as induced by ISCOM vaccine injection, is LB dependent, we used similar approaches as *in vitro*. Cross-presentation of the ISCOMs/OVA vaccine in *Adrp−/−* or *Igtp−/−* mice lacking functional LBs was hampered compared with wild-type mice (Fig. 6j; Supplementary Fig. 14a). Consistently, injecting the DGAT inhibitors Xanthohumol or A922500 significantly reduced cross-presentation (Supplementary Fig. 14b). Importantly, OVA K^b^ peptide presentation was intact in all cases, proving equal MHC-I levels and viability of the CD11c+ cells (Fig. 6j; Supplementary Fig. 14). CD11c+ cell percentage or activation status of these DCs was similar in these experiments (Supplementary Fig. 15). Finally, analysis of a mix of LB inhibitors in the *in situ* tumour ablation model confirmed that also here the induction of LBs was essential for the SBA-induced cross-presentation (Fig. 6k).

Collectively, these results uncover a causal connection between SBA-induced LBs and cross-presentation *in vivo*, and link for the first time LB formation in DCs to adjuvant-mediated vaccine efficiency.

## Discussion

SBAs generate strong Th1-driven cellular and humoral immune responses in various pre-clinical models and in vaccinated cancer patients^12^,^37^,^38^,^39^. How SBAs facilitate immune responses to vaccine antigens remains incompletely understood. We now show that SBAs specifically enhance antigen cross-presentation and the induction of LBs in the monocytic CD11b+ DC subset. Using genetic and pharmacological interference, we demonstrate that LB induction in DCs is causally related to the SBA-induced increase in antigen cross-presentation and subsequent T-cell activation.

Our sorting experiments identify monocytic CD11b+ DCs, as present in GM-CSF DC cultures *in vitro* and in LNs *in vivo,* as the only cells capable of reacting to SBAs by induction of cross-presentation and LB formation. Among all individual DC populations generated in *in vitro* Flt3-L cultures or isolated from naive spleens no SBA reactive population could be found, coinciding with the absence of migratory monocytic cells. Interestingly, GM-CSF exposure of the Flt3 DC cultures is required for the monocytic precursors present in these cultures to respond to SBA. This finding is in line with GM-CSF (Csf-2) acting either late in differentiation or activation of monocytic cells. A previous study by Wilson *et al*. showing SBA-induced cross-presentation in the LN migratory DC fraction confirms our results. However, this study also reported significant levels of cross-presentation in CD8α+ DCs. This discrepancy with our results could possibly be explained by background cross-presentation of non-formulated OVA antigen by CD8α+ DCs in their study. *In vivo* SBA vaccination furthermore will induce inflammation, influencing co-stimulatory signals on DCs and subsequent activation of the OT-I T cells used in the Wilson study. Our B3Z cells react co-stimulation independent which avoids such activation biases. Earlier studies in human DCs demonstrated that SBA vaccination with the cancer antigen NY-ESO-1 induces cross-presentation by myeloid DC, such as MoDC^51^. Our preliminary data show LB induction in these human moDCs generated in the presence of GM-CSF, linking SBA-induced cross-presentation and LB formation also in the human context.

Thus, SBAs act to induce potent cross-presentation and LB induction in monocytic CD11b+ DCs, a population of DCs distinct from the well-described CD8α+ cross-presenting DC. Together with the reported increase of CD11b+ DCs at SBA vaccination sites, this may well explain the efficient cross-priming of CD8+ T cells by SBA vaccines.

Despite the importance of cross-presentation in health and disease, the underlying molecular processes are still poorly understood. Studies so far indicate two main pathways: the ‘cytosolic' and the ‘vacuolar' pathway^52^. Low proteolytic activity of the endocytic pathway and endosomal stress resulting in ROS production by the NADPH oxidase complex 2 (NOX2), have shown to be imperative for efficient cross-presentation of CD8α+ DCs^41^. Our cross-presentation studies use a short time of SBA exposure (1 to 5 h), and in this timeframe no role for ROS could be detected. Our translocation studies show that SBAs facilitate endosomal escape, and that this is essential for cross-presentation. However, since not all pore-forming saponins show adjuvant activity, and SBA activity is critically dependent on DCs, other mechanistic elements must be involved as well. Here, the presence of LBs appears to be equally essential for cross-presentation, although LBs seem to be dispensable for endosomal escape of antigens, and vice versa. The observation that both SBA-triggered processes can be uncoupled at the level of the endosome makes it unlikely that LBs alter the phagosomal environment in any direct manner, as also put forward by Bougneres *et al*.^31^

Various types of (metabolic) stress can lead to the induction of LBs. TLR ligands were shown to increase glycolysis that fueled the *de novo* synthesis of fatty acids in LBs, essential for TLR ligand-induced DC activation^53^. Also other studies have correlated DC activation status with lipid content: When fatty acid synthesis of high-lipid-containing DCs was inhibited, these DCs lost their immunostimulatory phenotype^54^. Likewise, the presence of oxidized lipids in LBs was shown to hamper DC functionality and antigen presentation abilities^55^. This suggests that not only the absolute lipid content of LBs in DCs, but also the composition of the LBs involved can modulate immune responses. In our hands, feeding DCs fatty acids like oleic acid did induce LBs, but not cross-presentation (Supplementary Fig. 16). Finally, the presence of an established tumour (for example, before cryo-ablation) is sufficient to change the LB profile of tumour resident DCs/monocytes. This complicates a solid analysis of LB induction due to SBA administration following ablation. Together, these data imply that not all LBs are functionally equal, and that various LB types may be present at the same time, in the same cell. The fact that SBAs share the induction of LBs with certain TLR ligands places these adjuvants in a peculiar position somewhere in between the microbial and non-microbial adjuvants. Molecular analysis of the ability of SBAs to facilitate cross-presentation specifically in monocytic CD11b+ DCs via endosomal escape and LB induction will be key to understand SBA adjuvant activity.

IGTP, an IFN-related GTPase associated with LBs in DCs, was shown to be important in cross-presentation of OVA coated on large latex beads^31^. Deletion of IGTP resulted in reduced IFNγ-induced LB numbers, but it was less established what role IFNγ had in their cross-presentation assays. As our studies use OVA protein, which in itself is not easily cross-presented, a direct comparison with the use of latex beads is difficult, but one could speculate that the relatively large size of the beads leads to endosomal stress and possibly endosomal rupture. This putative common factor between SBA- and beads induced cross-presentation could implicate that LBs may become particularly important in forced types of cross-presentation.

The data presented here indicate an important role for IGTP and ADRP in the *in vivo* cross-presentation of soluble antigens after vaccination with SBAs. Whether this implicates direct involvement of these molecules, or that the effects are the indirect result of reduced LB functioning remains to be determined. To find causal connections at a more detailed level, sub-organelle/molecular studies using purified LBs from differentially treated immune cells are required. An intriguing finding is that GM-CSF-cultured DCs and CD11b+ LN DCs are uniquely able to cross-present antigen upon SBA exposure. It will be important to explore the molecular basis for this DC subset specificity, and how this can be linked to LB formation, antigen cross-presentation and immune activation.

The SBAs used in our study do not contain any known PRR ligand, and consistent with this they fail to upregulate co-stimulatory molecules on DCs *in vitro* (Fig. 2b and ref. 17). Moreover, cross-presentation was not hampered in *Tlr4−/−*, or *Myd88−/−* cells (Fig. 2c). Analysis of cells deficient in type I IFN or the NLRP3 inflammasome showed that these pathways were not involved either. Interestingly, others repeatedly reported an *in vivo* dependency of successful SBA vaccination on MyD88, while TLR4 and IL-1R seemed dispensable^16^,^17^. This suggests that effective vaccination depends on the *in vivo* release of endogenous danger signals or the involvement of indirect cytokine signalling. IL-18 signalling is involved in indirect NK cell and CTL activation following vaccination with SBAs^16^, which could partly explain the dependency on MyD88, being an adaptor for the IL18 receptor. Importantly, in this study MyD88 deficiency had no *in vivo* effect on maturation or vaccine antigen cross-presentation by DCs^16^. This research connects the initial innate cytokine-cascade with the adaptive cellular response, but leaves room for MyD88/TLR independent mechanisms of DC activation and cross-presentation with an alternative molecular basis.

Cancer vaccines evaluated to date mostly fail to change and overcome the immunosuppressive microenvironment present in many solid tumours. Current strategies using SBAs and cancer antigens (for example, NY-ESO-1) have proven to not only induce antibody responses, but also robust CD8+ T-cell expansion^17^,^37^. Nevertheless, vaccinations with single injections of SBA-peptide compositions yielded only modest survival benefit for patients with established tumours^56^. Interestingly, when these vaccines were combined with TLR ligands like CpG-ODN^57^,^58^, or followed up by a boosting regimen^59^, beneficial correlates of immunological protection could greatly be improved. We have previously shown that *in situ* tumour ablation can provide a source of (neo)-antigens for the generation of anti-tumour immune responses, provided that additional immune stimulation is given^33^. SBAs appeared to be superior in creating synergy between the ablation and adjuvant-induced immune effects, inducing long-lasting tumour-directed immunity^11^. This makes tumour ablation with local co-administration of SBAs a promising treatment strategy to explore further in clinical settings^60^. Future studies will have to show whether combinations of SBAs and TLR ligands are also effective combinations in ablation settings, and it will be appealing to correlate the clinical outcome to the presence and composition of LBs.

Collectively, our data shows that SBA-induced LBs are elementary in the mechanism of SBA adjuvanticity. Understanding the LB-immune connection in SBA vaccination will help to design new adjuvants for improvement of (cancer) vaccines, aiming to enhance cross-presentation and the subsequent induction of potent anti-tumour CTL responses.

## Methods

### Mice

*Igtp−/−* and *Adrp−/−* mice were a kind gift of S. Amigorena (Institut Curie, Paris), and have been described before^61^,^62^. *Myd88−/−*, *Tlr4−/−* and *Trif−/−* mice were a kind gift of S. Akira (Osaka University, Japan). *Nlrp3−/−* bone-marrow was a kind gift of L. Joosten and T. Kanneganti (Nijmegen, the Netherlands). *Gp91phox−/−* bone-marrow was a kind gift of A. Schreiber (Berlin, Germany). All experiments using knockouts were performed with +/+ littermates on C57Bl/6JRccHsd background, (>8 crossings). For *Igtp−/−* and *Adrp−/−* mice, a common +/+ littermate was used (for clarity in those experiments referred to as wild type). All experiments without knockouts were performed using female wild-type C57Bl/6JRccHsd mice purchased from Charles River (Sulzfeld, Germany), and were used as 7–11 weeks old animals. C57Bl/6 OT-IxCD90.1^+^ (Thy-1.1) and C57Bl/6 OT-II mice were held in the Nijmegen Animal facility. All mice were maintained on standard lab chow and sterile water, and housed under specific pathogen-free conditions in IVC cages at the Nijmegen Animal facility. All animal experiments were approved by the Animal Experimental Committee of the Radboud UMC, and were performed in accordance with institutional, national and European guidelines.

### Cells

Primary cultures of bone-marrow-derived dendritic cells (BMDCs) were generated by culturing total bone marrow cells in complete medium (Roswell Park Memorial Institute (RPMI) medium (Gibco), 10% fetal calf serum (FCS), 1% L-alanyl-L-glutamine, 0.1% β-mercapto-ethanol and 1% antibiotics/antimycotics (AA, Gibco)), containing 20 ng ml^−1^ granulocyte-macrophage colony stimulating factor (GM-CSF DCs, culture 7 days), or containing 200 ng ml^−1^ Fms-related tyrosine kinase 3 ligand (Flt3-L DCs, culture 10 days), as adapted from previously described protocols^63^,^64^. GM-CSF and Flt3-L were purchased from Peprotech (Rocky Hill, CT, USA). In some experiments the Flt3-L cultures were supplemented with 20 ng ml^−1^ GM-CSF for the indicated periods. *Ex vivo* DCs were isolated from pooled superficial inguinal and axillary lymph nodes, or spleens, according to previously published protocols, as indicated. For some of these experiments, mice were injected before with a B16-F10 melanoma expressing Flt3-L to expand endogenous DC pools, as described before^34^,^45^. B16-Flt3-L cells were cultured in Iscove's Modified Dulbecco's Medium (IMDM) supplemented with 5% FCS and 1% AA, and 5 × 10^6^ cells were injected s.c. to establish Flt3-L-producing tumours in 9–12 days. Sorting of DC populations was performed after incubation in serum-free medium containing collagenase (Worthington, Lakewood, NJ, USA) and DNAse I (Roche) at 37 °C, later supplemented with 1 mM EDTA. For isolation of total CD11c+ fractions, a magnetic bead kit was used according to manufacturer's instructions (Clone N418, Miltenyi Biotech, Bergisch Gladbach, Germany). For isolation of DC subsets, total cell suspensions were sorted on a FACS Aria system (BD biosciences), based on the markers as indicated in the figures. Purity (>98%) and additional markers were confirmed using a FACS Cyan system (Beckman Coulter). CD8+ T cells specific for the OVA257-264 SIINFEKL peptide in an H-2K^b^ MHC-I context, or CD4+ T cells specific for the OVA323-339 peptide in an H-2IA^b^ MHC-II context, were obtained from pooled spleen and lymph nodes of OT-IxCD90.1 or OT-II transgenic mice, respectively. Before use, both cell types were purified by magnetic bead sorting (positive selection on CD8, Miltenyi). B3Z cells were cultured in Iscove's Modified Dulbecco's Medium (IMDM) supplemented with 5% FCS, 0.1% β-mercapto-ethanol, 1% antibiotics/antimycotics, 500 μg ml^−1^ hygromycin. The murine melanoma cell line B16F10 (ATCC) was cultured in complete medium (MEM, 5% fetal bovine serum (Greiner Bio-one), 100 U ml^−1^ penicillin G sodium and 100 μg ml^−1^ streptomycin (Pen/Strep), MEM sodium pyruvate (1 mM), NaHCO_3_, MEM vitamins, MEM non-essential amino acids (all from Gibco), 20 μM beta-mercaptoethanol). OVA-transfected B16F10 (B16OVA, clone MO5) was cultured in complete medium supplemented with 30 μg ml^−1^ hygromycin and 1 mg ml^−1^ G418. B6MECsigOVA cells are transfected with murine B7.1, H-2Kb, and a construct expressing an ER targeting signal sequence, followed by the OVA257–264 CTL epitope SIINFEKL (original name B6-B7.1-sigOVA^65^). These cells were cultured in IMDM with 5% FCS, 1% L-alanyl-L-glutamine, 0.1% β-mercapto-ethanol, 1% antibiotics/antimycotics, 100 μg ml^−1^ Hygromycin, and 100 μg ml^−1^ G418. All cell-lines in our study were tested mycoplasma negative.

### Antibodies and reagents

For FACS experiments the following antibodies (clone name in brackets) conjugated to various fluorophores were used, together with corresponding isotypes. All antibodies were used in a 1:400 dilution in PBA, unless otherwise stated after the clone name. Anti-CD206 (C068C2; 1:100) (AbD Serotec); anti-CD8α (53-6.7), anti-CD8β.2 (53-5.8; 1:100), anti-CD11c (HL3), anti-CD69 (H1.2F3), anti-CD135 (A2F10.1; 1:100), anti-CD172 (P84), anti-Ly6C (AL21), anti-B220 (RA3-6B2; 1:100) (all BD Biosciences); anti-CD14 (Sa2-8; 1:800), anti-CD90.1-Biotin (HIS51), anti-CD115 (AFS98; 1:200), anti-I-A^b^/I-E^b^ (M5/114.15.2; 1:800), anti-SiglecH (eBio440c; 1:100) (all eBiosciences); anti-CD4 (RM4-5), anti-CD11b (M1/70), anti-CD11c (N418), anti-CD24 (M1/69), anti-CD44 (IM7; 1:1,000), anti-CD80 (16-10A1), anti-CD86 (GL1), anti-CD103 (2E7; 1:800), anti-H-2K^b^/D^b^ (28-8-6) (all Biolegend); streptavidin-Alexa488, -555, -647 (Invitrogen; 1:800). All stainings were preceded by blocking Fc receptors using anti-CD16/CD32 antibodies (BD; 1:800). For confocal experiments the following antibodies/reagents were used: chicken anti-mouse/human ADRP (Abcam; 5 μg ml^−1^), Goat anti-chicken IgY-DyLight^549^ (Abcam; 5 μg ml^−1^), Bodipy^493-503^ dye (Invitrogen), and DAPI dye (Sigma, St Louis, MO, USA). Various assays were done in the presence of the following inhibitors or ROS scavenging compounds: diphenyleneiodonium chloride (DPI) and Bafilomycin A1 were purchased from EMD Millipore (Billerica, MA, USA); acetovanillone (Apocynin), L-ascorbic acid (Ascorbic acid), 6-hydroxy-2,5,7,8-tetramethylchromane-2-carboxylic acid (Trolox, a vitamin E analog), N-acetyl- L-cysteine (LNAC), DGAT-1 specific inhibitor A922500, chloroquine, and epoxomicin all were from Sigma; DGAT 1/2 inhibitor xanthohumol was from Tocris Biosciences (Bristol, UK); Triacsin C was from Enzo Biochem (Farmingdale, NY, USA); 5-Tetradecyloxy-2-Furoic Acid (TOFA), N-([3-(Aminomethyl)phenyl]methyl)ethanimidamide dihydrochloride (1,400 W), and 2-Cyano-3,12-dioxo-oleana-1,9(11)-dien-28-oic acid methyl ester, bardoxolone Methyl (CDDO-me) were from Cayman Chemical (Ann Arbor, USA); Murine IFN-gamma was from Peprotech. Water-soluble cholesterol, PMA and cis-9-octadecenoic acid (Oleic acid) were from Sigma. Ovalbumin conjugated to various alexa dyes was obtained from Invitrogen, double quenched (DQ)-OVA was purchased from ThermoFisher Scientific. Cytochrome C from equine hearth was purchased from Sigma. Saporin from Saponaria officinalis L (a kind gift from M. Thakur), is a type I RIP that is internalized by receptor-independent endocytosis and accumulates in late endosomes and lysosomes^66^. Type I RIPs and cytochrome C can only exhibit cytotoxicity if they can efficiently escape from the endo/lysosomes.

### Adjuvants

CpG 1668 (5′-TCCATGACGTTCCTGATGCT-3′) with total phosphorothioate-modified backbone was purchased from Sigma. The following non-microbial adjuvants were used (all MSD Animal Health, Boxmeer, the Netherlands): 45/55 w/w water-in-oil emulsion using Miglyol 840 and 0.5% w/w Arlacel P135; MF59 oil-in-water emulsion using squalene; Aluminum phosphate 0.75% in phosphate buffer. All adjuvants were tested in a large titration in the B3Z assays. As saponin-based adjuvants (SBAs) the *Quillaja saponaria*-derived adjuvants Matrix C ISCOMs (MSD) and Quil A saponin (MSD) as indicated were used. As a control, *Quillaja saponaria*-derived saponins not purified for immunoactive fractions were obtained from Sigma (‘crude saponin'). Next to the proprietary made Matrix C, ISCOM structures were used produced via an open access method^40^. In short, three immunoactive saponin fractions ‘Supersap (SS)', ‘Vaxsap (VX)' and ‘Fraction C (FC)' were obtained from Desert King International (San Diego, US). Fifty milligram SS or FC saponin was slowly dissolved in milli-Q water to obtain 100 mg ml^−1^. Next, 1 gram n-(D-glucityl)-n-methyldecanamide-n-decanoyl-n-met (Mega10) was dissolved in MilliQ to obtain a 20% (w/w) solution. This solution was used to dissolve 50 mg cholesterol (Sigma) and 50 mg phosphatidyl choline S (Lipoid, Germany). At a temperature of 25.5 °C, the saponins were added to 1 ml of chol/pc mixture. After adjusting the cholesterol concentration to 1 mg ml^−1^ by adding PBS, the mixture was incubated overnight in a shaker at 25.5 °C, and subsequently dialysed against PBS. ISCOMs structures of ±40 nm were confirmed by bringing the solution onto a carbon-coated formvar plasm grid, which was activated with a 1% uranylacetate solution, and analysed using a Versa 3D electron microscope. Saponin content was analysed with an HPLC reversed phase system (Thermo RSLC 3000), using a Supersphere 60 RP select B column, and an acetonitrile/water/TFA gradient.

### Flow cytometry

Phenotypic characterization of DCs and T cells was performed using standard antibody staining protocols, and analysis was done using a FACS Cyan (Beckman Coulter) or a FACS Verse system (BD). All stainings were preceded by Fc-receptor blockade using anti CD16/32 antibodies. APC maturation status was evaluated using CD80, IA^b^ or K^b^ staining after gating on CD11c+ cells. Receptor expression of CD206 (mannose receptor), K^b^ levels, OVA uptake and degradation were used to prove equal abilities of various DCs to internalize, process and present antigens. For OVA uptake and degradation assays, cells were incubated for 5 h with 0.25 mg ml^−1^ OVA coupled to Alexa647 (Invitrogen) or with 1.0 μg ml^−1^ double quenched DQ-OVA (which fluoresces once degraded in the endolysosomal system).

### B3Z and OT-I cross-presentation assays

For the cross-presentation assays, 80 × 10^3^ cells (*in vitro* BMDCs) or 150 × 10^3^ cells (*ex vivo* DCs) were pulsed with endotoxin-free chicken egg ovalbumin (OVA, Hyglos GmbH, Germany) in the presence of the indicated adjuvants or inhibitors. Typically, 400 ng ml^−1^ SBAs were added, and 80 μg ml^−1^ OVA, unless indicated otherwise. After 5 h of incubation at 37 °C, cells were washed and cultured for 18 h with 80 × 10^3^ B3Z cells. B3Z cells and positive control B6-B7.1-sigOVA cells were cultured as described elsewhere^67^. As a control for cell-viability and/or MHC-I expression levels, DCs were washed 30 min before adding the B3Z cells and pulsed with 5 ng ml^−1^ peptides. Peptides (OVA: SIINFEKL, HPV: RAHYNIVTF, Ad-E1A: SGPSNTPPEI) were obtained from J.W. Drijfhout (LUMC, Leiden, the Netherlands). In one experiment standard PEG-ylated PLGA nanoparticles formulated with OVA were used, which were produced in our lab as described elsewhere^68^. The amount of particles was adjusted so that the wells contained an OVA end-concentration of 30 μg ml^−1^. The presentation by DCs of SIINFEKL in H-2K^b^ results in production of β-galactosidase by the B3Z cells^65^, which can be detected by adding 0.15 mM chlorophenolred-h-D-galactopyranoside (Calbiochem), 9 mM MgCl_2_, 0.125% NP40, and 100 mM β-mercaptoethanol in PBS. Plates were incubated for 2 to 5 h at 37 °C and absorbance values were measured using a photospectrometer (595 nm).

For the OT-I/II cross-presentation assays 25 × 10^3^ DCs were plated and treated as indicated above. Purified OT-I or OT-II cells (CD8 or CD4 MACS beads) were labelled with cell-proliferation dyes CFSE or PBSE, according to the manufacturer's instructions (Invitrogen), and 50 × 10^3^ cells were incubated with the DCs for up to 72 h. After one day of incubation, cells were stained with CD90.1, CD8, and the early activation marker CD69. After 3 days, the late stage activation marker CD44 and CFSE/PBSE dilution was analysed instead. All T-cell analyses were performed after double gating on CD8β and CD90.1. Cells were measured using a FACS Cyan (Beckman Coulter) or a FACS Verse system (BD).

### Lipid body stainings

Lipid bodies were visualized by CLSM or FACS. For confocal microscopy, 250 × 10^3^ DCs were plated for 45 min at 37 °C on glass coverslips coated overnight with 20 μg ml^−1^ fibronectin (Sigma). After washing with PBS, cells were exposed to the indicated treatment conditions in serum-free medium for 5 h. Typically, 400 ng ml^−1^ SBAs were used, unless otherwise indicated. Next, cells were fixed in 4% PFA solution in PBS for 15 min. For co-staining for ADRP, fixed cells were permeabilized with 0.1% Saponin (Sigma) in PBA and incubated with primary antibodies in PBS plus 0.2% BSA/0.05% saponin for 1 h. Next, cells were washed and incubated with anti-chicken IgY secondary antibody conjugated to Dyelight549 (Abcam) for 45 min. LBs were visualized by adding Bodipy^493-503^ probe (Invitrogen) at 7 μg ml^−1^ for 10 min. Nuclear counterstaining was performed with DAPI dye according to the manufacturer's instructions. After washing, coverslips were mounted onto the slides using standard Mowiol sealing. Images were acquired using a Olympus FV1000 confocal laser scanning microscope (Olympus, Tokyo, Japan) with a 60 × 1.35 NA Oil immersion objective. Merged images were generated using ImageJ software (NIH, Bethesda, Maryland). For the counting of LB numbers around 15 images were taken at random spots in the sample, which were analysed using FIJI software. Nuclear defragmentation as detected by the DAPI stain was used to exclude apoptotic cells (<2%), and only cells which were entirely visible in the image were taken along for counting. LB numbers were counted manually, yielding each time data of 90–150 cells. For FACS analysis, cells were exposed to the indicated treatment conditions in 96 well plates for 5 h, washed, and fixated for 10 min with 1% PFA. Next, cells were permeabilized using PermWash according to the manufacturer's instructions (eBioscience). Cells were then incubated with 10 μg ml^−1^ Bodipy^493-503^ for 30 min at 4 °C. After washes, Bodipy fluorescence was detected in the FL1 (488: 530/40) channel on a FACS Cyan system (Beckman Coulter). The Bodipy signal integrates info from both the number, size and intensity of LBs (validated by Gocze *et al*.^69^).

### ROS detection

The production of total cellular ROS was detected using DHR123 and H2DCFDA. 150 × 10^3^ GM-CSF cultured BMDCs were plated in serum-free medium in 96 wells round bottom plates (Costar). Cells were pretreated for 10 min with 0.5 or 50 μM dihydrorhodamine 123 (DHR123, Sigma), after which the following compounds were added: 400 ng ml^−1^ ISCOMs or 1 μg ml^−1^ phorbol 12-myristate 13-acetate (PMA, Sigma). After 15 or 45 min incubation, DHR staining was directly measured by FACS. Similarly, ROS was detected using CM-H2DCFDA (Invitrogen). For this, cells were washed with HBSS containing magnesium and calcium, after which they were incubated for 45 min with 10 μM H2DCFDA and the above stimuli dissolved in HBSS buffer.

### MTT assays

GM-DCs were seeded in flat-bottom 96-well plates (Costar) at 170 × 10^3^ cells per well and cultured overnight to adhere. Next, they were exposed to the indicated compounds for 5 h (ISCOMs: 800 ng ml^−1^, Saporin: 1 nM, Cytochrome C: 2.5 mg ml^−1^). After cells were washed and rested o/n, their metabolic activity/viability was measured in a standard MTT assay. In short, 10 μl MTT reagent (4 mg ml^−1^) (Sigma) was added in medium. Plates were incubated for 1–4 h at 37 °C after which supernatant was removed and 100 μl lysis buffer (0.5% SDS, 4% 1 N HCl, and 3.5% Milli-Q in isopropanol) was added. After 15 min. of incubation, absorbance was measured at 595 nm. Metabolic activity versus control was calculated as (treatment—blank)/(control—blank) × 100%. Triplicate wells for each concentration were performed.

### *In vivo* procedures

The *in vivo* effects of ISCOMs on cross-presentation and LB induction were analysed in wild-type, ADRP/IGTP knockouts or their +/+ controls (*n*=6). For this, 300 μg endofree OVA protein was injected s.c. on the femur, together with 30 μg ISCOMs and/or LB inhibitors. LB inhibitors (or vehicle) were injected twice: 4 h before, and together with the OVA/ISCOMs injection (Xanthohumol: 500 μg per injection, A922500: 150 μg per injection). To control for MHC-I levels and cell viability, 40 μg OVA K^b^ peptide was injected in control mice. 12 h later, draining lymph nodes were harvested from which a single cell suspension was made using collagenase/DNAse containing serum-free medium. CD11c+ fractions were isolated using magnetic bead sorting on CD11c according to the manufacturer's instructions. Next, these cells were processed for the B3Z/OT-I cross-presentation assays and LB stainings similarly as in the *in vitro* assays, with one exception: the co-incubation of DCs with B3Z cells was longer (42 instead of 18 h), to allow the B3Z cells more time to produce β-galactosidase.

The cryo-ablation model, in which established tumours are destroyed by freezing has been described in more detail in the following articles^11^,^33^,^34^. In short, B16F10 melanomas or B16F10 cells expressing ovalbumin (B16OVA) growing s.c. on the femur with a diameter of 5–8 mm were treated by macroscopically freezing the entire tumour using a liquid nitrogen-cooled CryoPro Maxi device (Cortex technologies, Hadsund, Denmark). Directly before ablation, mice received an intratumoural booster shot with 20 μg OVA, whenever indicated. Directly after ablation, 30 μg ISCOMs or 50 μg CpG, as indicated, was injected in the peri-tumoural area.

For tetramer detection of specific T cells in vaccinated or ablated mice, mice were injected s.c. on the right femur with 20 μg endofree OVA and/or 30 μg ISCOMs, or ablated as described above. Ten days later the draining lymph nodes (sup. ing.) were harvested from which a single cell suspension was made using collagenase/DNAse supplemented medium. First, cells were stained for anti CD8β.2 on ice, after which OVA K^b^ specific tetramers (Pelimers; Sanquin, Amsterdam, the Netherlands) were used to stain for OVA-specific T cells. Staining was done according to manufacturer's instructions, at 37 °C, and analysed on a FACS Calibur system (BD). No randomization of animals or blinding was performed in these *in vivo* experiments during the treatment phase, but sample analysis was performed blinded by coding samples.

### Statistical analysis

Depending on the experimental layout, Student's *T* test, one-way ANOVA, or two-way ANOVA was performed with *post hoc* Tukey or Bonferroni tests, as indicated in the figure legends. For *in vivo* data showing biological replicates sample sizes were computed using a-priori poweranalyses for ANOVA, using a power of 0.8 and alpha 0.05. Differences were considered significant when *P* values were smaller than 0.05. In all figures, results are expressed as mean values from triplicates with s.e.m., unless indicated otherwise, while the following symbols were used: *P<0.05; **P<0.01; ***P<0.001; n.s., *P*>0.05; ND=not determined.

### Data availability

The data that support the findings of this study are available from the corresponding author upon request.

## Additional information

**How to cite this article:** den Brok, M. H. *et al*. Saponin-based adjuvants induce cross-presentation in dendritic cells by intracellular lipid body formation. *Nat. Commun.* **7,** 13324 doi: 10.1038/ncomms13324 (2016).

**Publisher's note:** Springer Nature remains neutral with regard to jurisdictional claims in published maps and institutional affiliations.

## Supplementary Material

Supplementary Information Supplementary Figures 1-16 and Supplementary Reference
