# Supplementary material for: Saponin-based adjuvants induce cross-presentation in dendritic cells by intracellular lipid body formation
Source: Nat Commun. 2016 Nov 7;7:13324. doi: 10.1038/ncomms13324 (PMC5103066; doi:10.1038/ncomms13324)
Supplement: Supplementary Information — Supplementary Figures 1-16 and Supplementary Reference [file ncomms13324-s1.pdf]

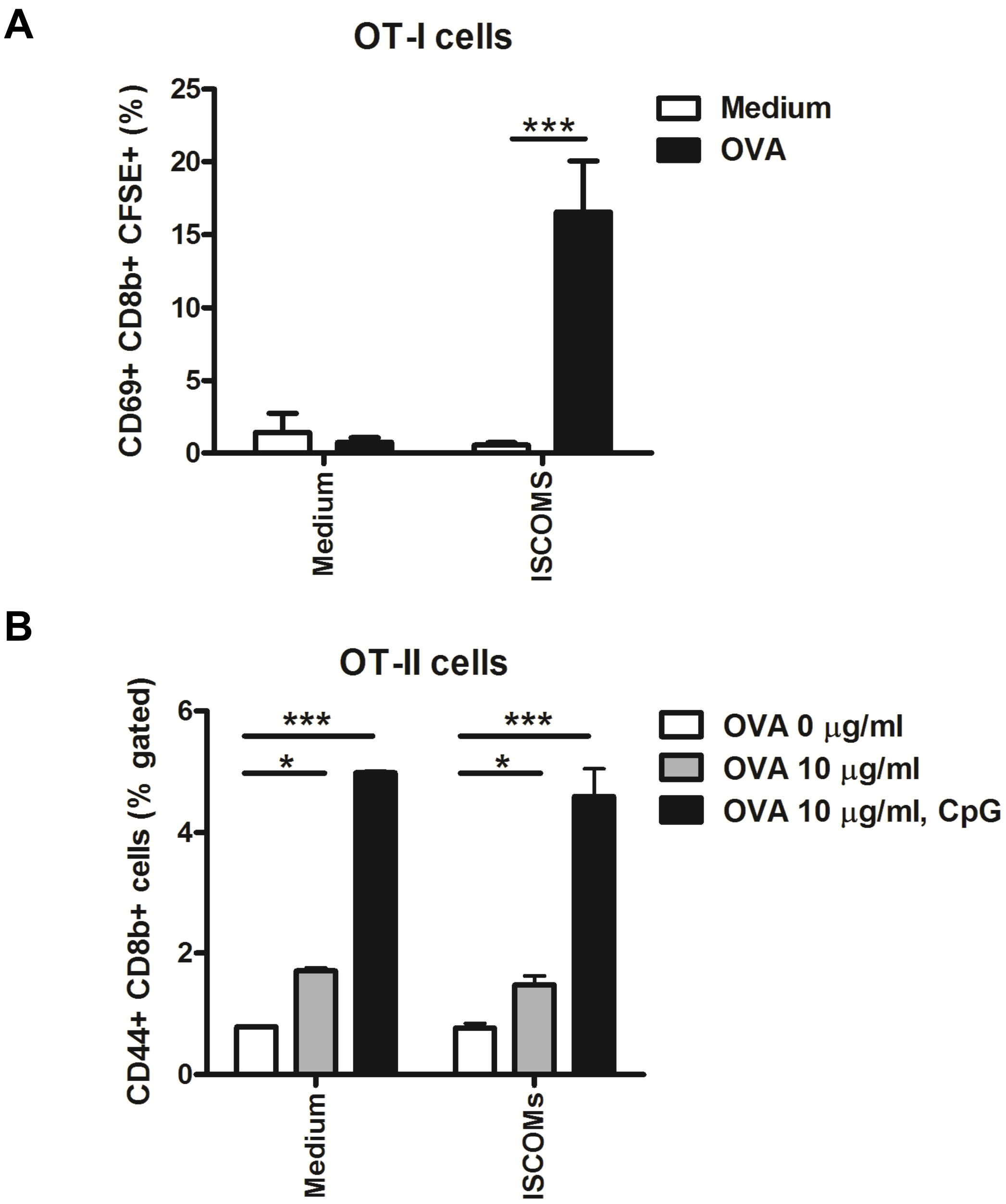

Supplementary figure 1. ISCOM adjuvant enhances MHC-I, but not MHC-II presentation of exogenous antigens. (A-B) *In vitro* OT-I and OT-II antigen presentation assays. GM-CSF-cultured BMDCs were exposed to the indicated compounds for 5 hrs, washed, and co-cultured with CFSE-labeled OT-I or OT-II cells. After one day, the expression of the early activation marker CD69 was measured on CD8β-gated, CFSE+ OT-I cells (A). Data depict mean percentages of CD69/CD8β/CFSE positive cells. After three days, the expression of the late activation marker CD44 and dilution of CFSE was measured on the OT-II cells (B). Data depict mean percentages of CD44 positive/CFSE low cells. The co-stimulation dependency of OT-I/II cells is illustrated by the addition of 0.1 μg/ml CpG. Statistical analyses were done using two way ANOVA with post hoc Bonferroni tests.

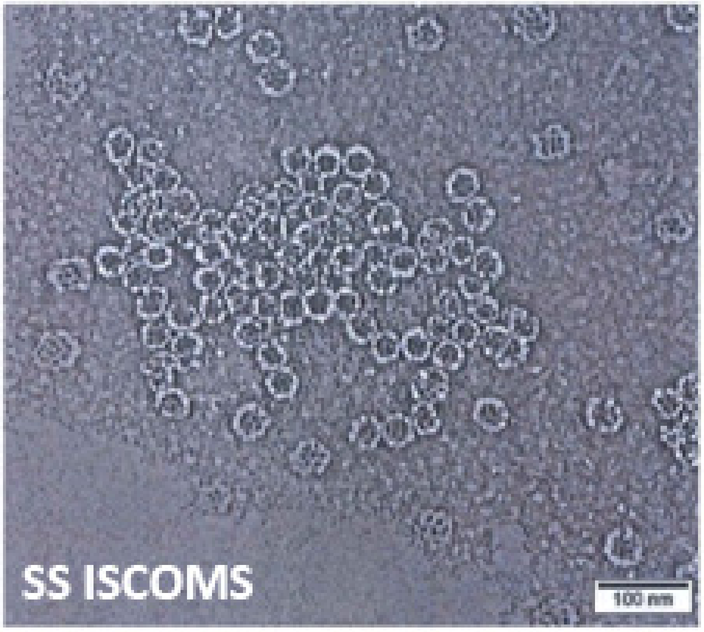

Supplementary figure 2. 'Open Access' ISCOM adjuvants have the correct shape and size. EM picture showing correct size (+/- 40 nm) and shape (cage-like round) of ISCOM structures made via an 'open access method' using SS saponin. Scale bar: 100 nm

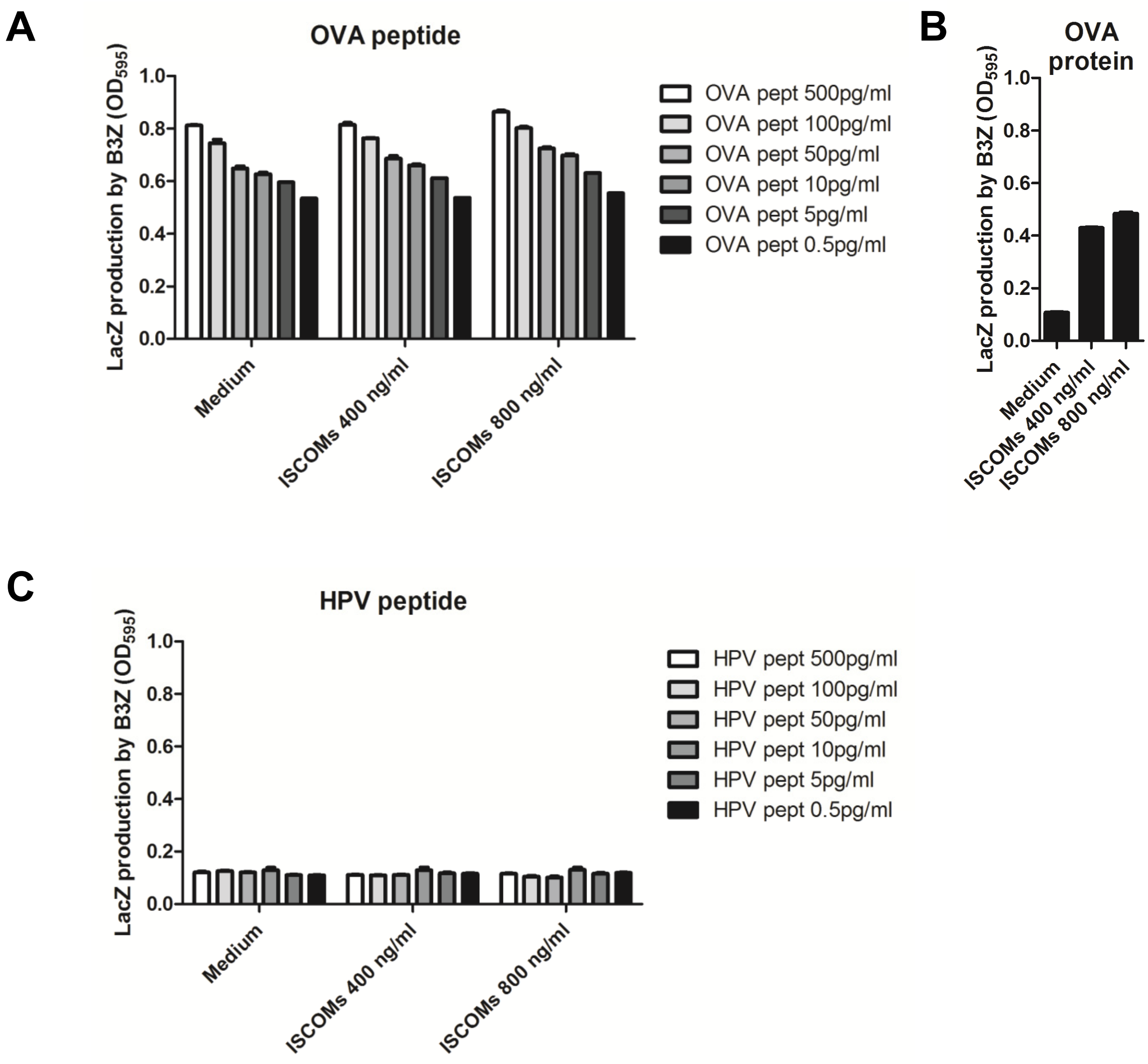

Supplementary figure 3. B3Z reaction to externally pulsed peptide dilutes normally and is not influenced by SBA addition. *In vitro* cross-presentation assay using B3Z cells. GM-CSF BMDCs were exposed for 5 hrs to ISCOMs (400 or 800 ng/ml) or medium. In this period, cells received medium (A and C) or 80 µg/ml OVA protein (B). After washing, cells were externally pulsed for 30 minutes with the indicated amounts of OVA or HPV K^b peptide. After washing, cells were incubated o/n with the B3Z cells. Data represent single values in titration.

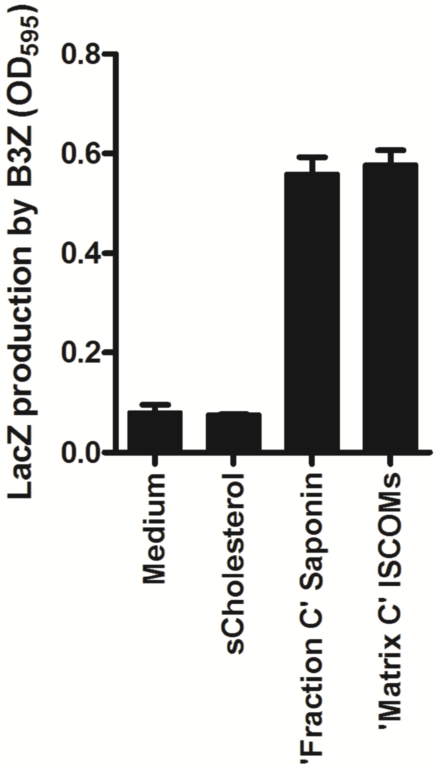

Supplementary figure 4. Saponins in the ISCOM structures are the active component inducing cross-presentation. Quantification of cross-presentation in GM-CSF BMDCs following 5 hrs exposure to solubilized cholesterol, ‘Fraction C’ saponin, or ‘Matrix C’ ISCOMs (made with Fraction C saponin) (all 400 ng/ml). Other components of ISCOMs are water-insoluble. Similar data were obtained in two independent experiments.

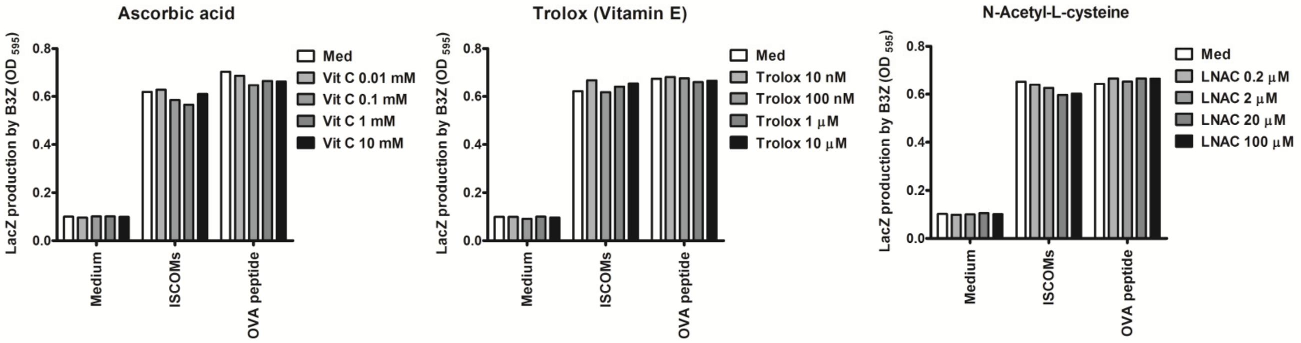

Supplementary figure 5. SBA-induced cross-presentation is not caused by endosomal ROS. *In vitro* cross-presentation assay in the presence of the indicated amounts of three ROS scavengers. Compounds were added during the 5 hr exposure period to ISCOMs. Data represent single values in titration. Similar data were obtained in two independent experiments.

A

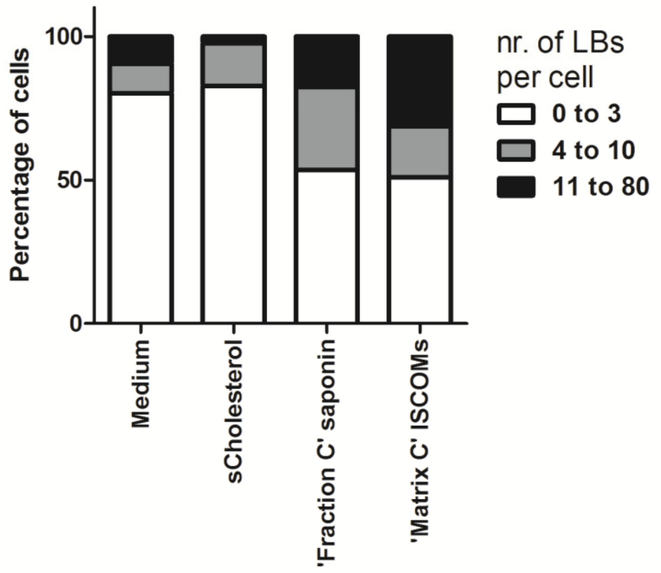

B

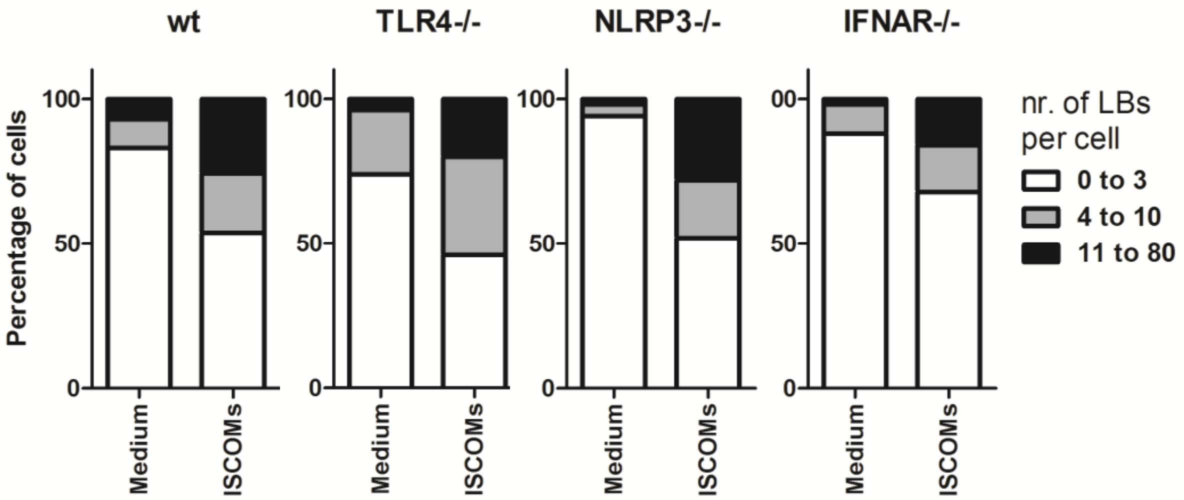

Supplementary figure 6. Saponins in the ISCOM structures are the active component inducing LBs, and LB induction is normal in various knockout DCs. (A) Quantification of LBs in GM-CSF BMDCs following 5 hrs exposure to solubilized cholesterol, ‘Fraction C’ saponin, or ‘Matrix C’ ISCOMs (made with Fraction C saponin) (all 400 ng/ml). Other components of ISCOMs are water-insoluble. Similar data were obtained in two independent experiments. (B) LB quantification in GM-CSF BMDCs from wild-type, TLR4-/-, NLRP3-/-, or type I IFN receptor-/- mice.

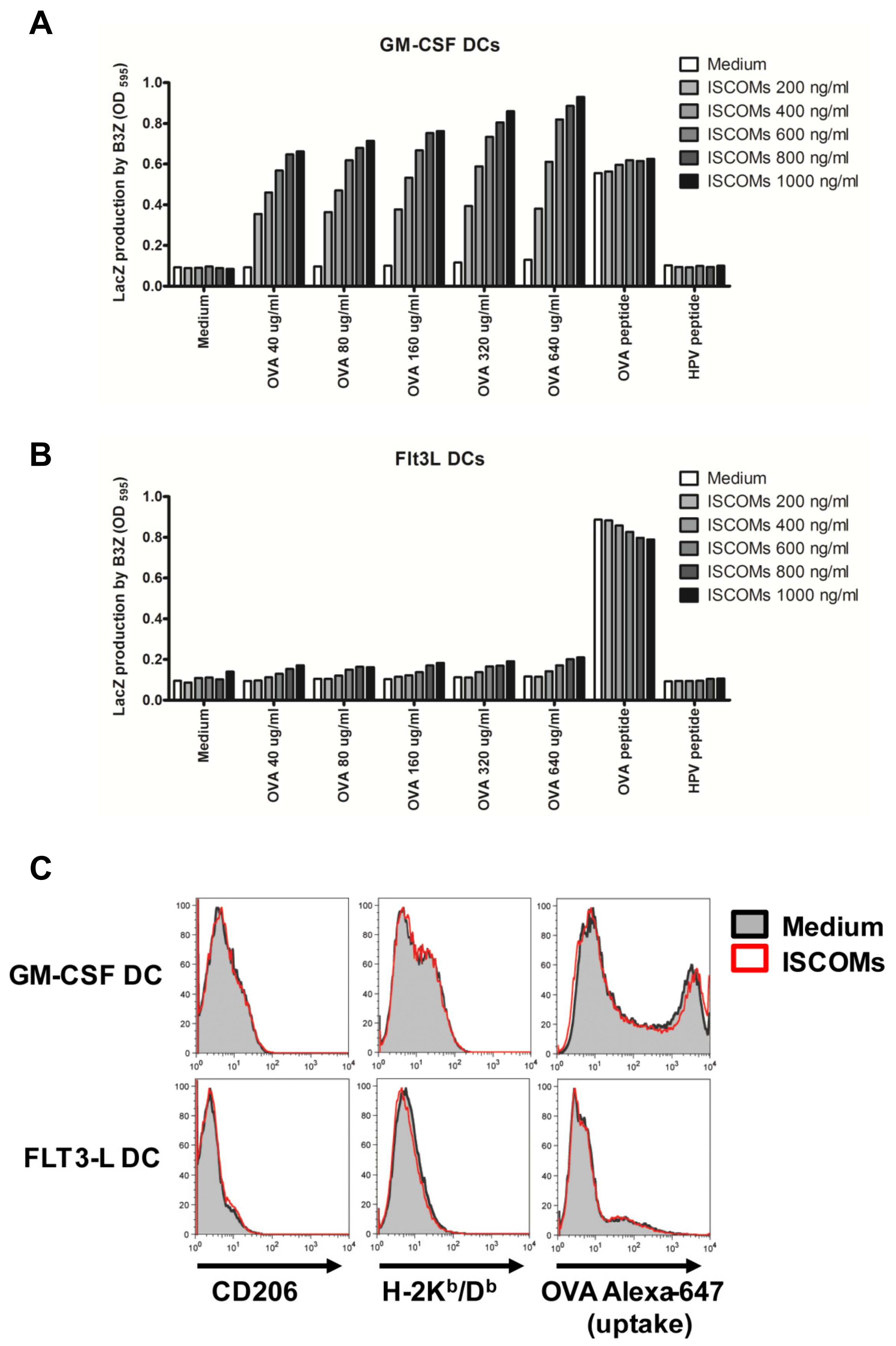

Supplementary figure 7: legends on next page

Supplementary figure 7. ISCOM adjuvant induces cross-presentation only in GM-CSF-cultured BMDCs. (A and B) *In vitro* cross-presentation after 5 hrs exposure of GM-CSF BMDCs or Flt3-L BMDCs to wide concentration ranges of OVA and ISCOMs. External peptide pulsing was used to control for viability and/or MHC-I levels. Data represent single values in titration. Similar results were obtained in two independent experiments. (C) FACS analysis of GM-CSF BMDCs or Flt3-L BMDCs. Cells were exposed for 5 hrs to medium, ISCOMs or 0.25 µg/ml OVA coupled to the fluorophore Alexa647. Next, cells were processed for FACS stainings with anti CD206 (mannose receptor), or anti MHC-I antibodies. The filled black lines show the medium-treated cells, while the open red lines are the ISCOM treated samples.

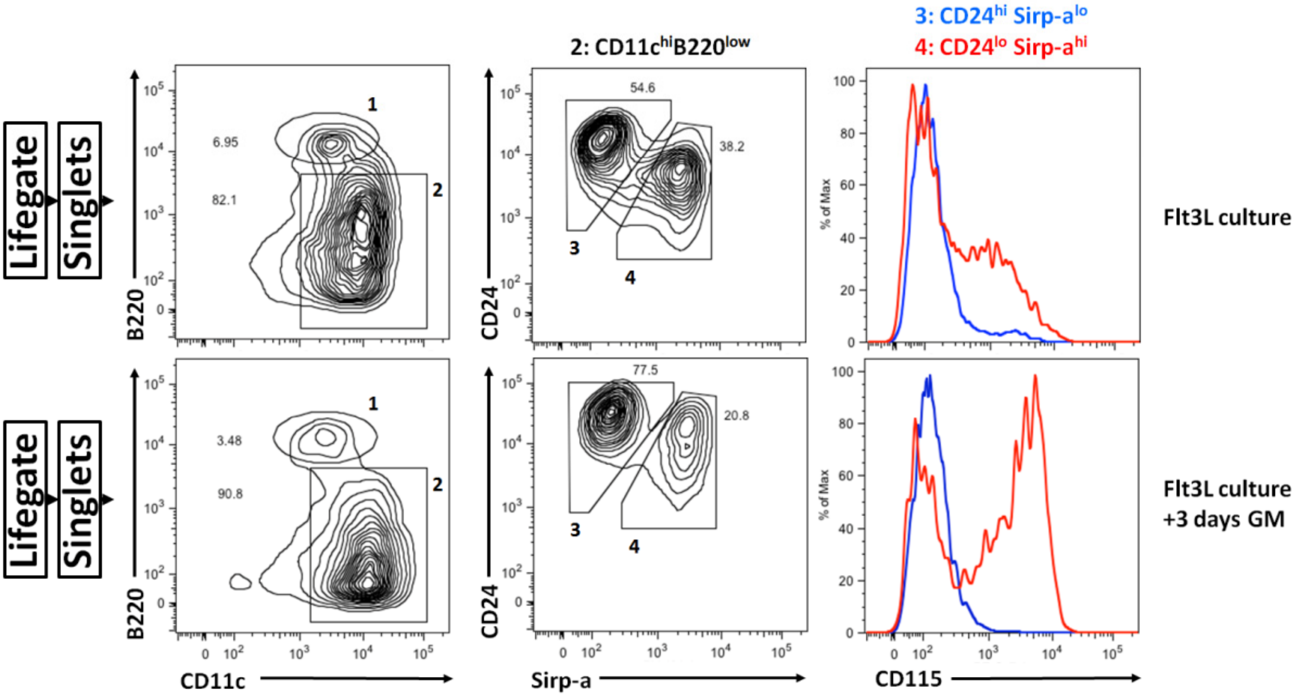

Supplementary figure 8. Gating strategy of Flt3-L cultures exposed to GM-CSF for an additional 72 hrs. Corresponding to figure 4I (left panel) the entire day 10 Flt3-L culture was supplemented with GM-CSF for another 72 hr, after which the resulting populations were sorted (Gate 1 pDCs, 3 CD8α+ DCs, and gate 4 CD11b+ DCs). Note that with GM-CSF, CD8α+ DCs increase at the expense of pDCs and CD11b+ DCs, similar as previously reported in Mayer et al[1]. Right panel shows additional staining for the monocytic marker CD115.

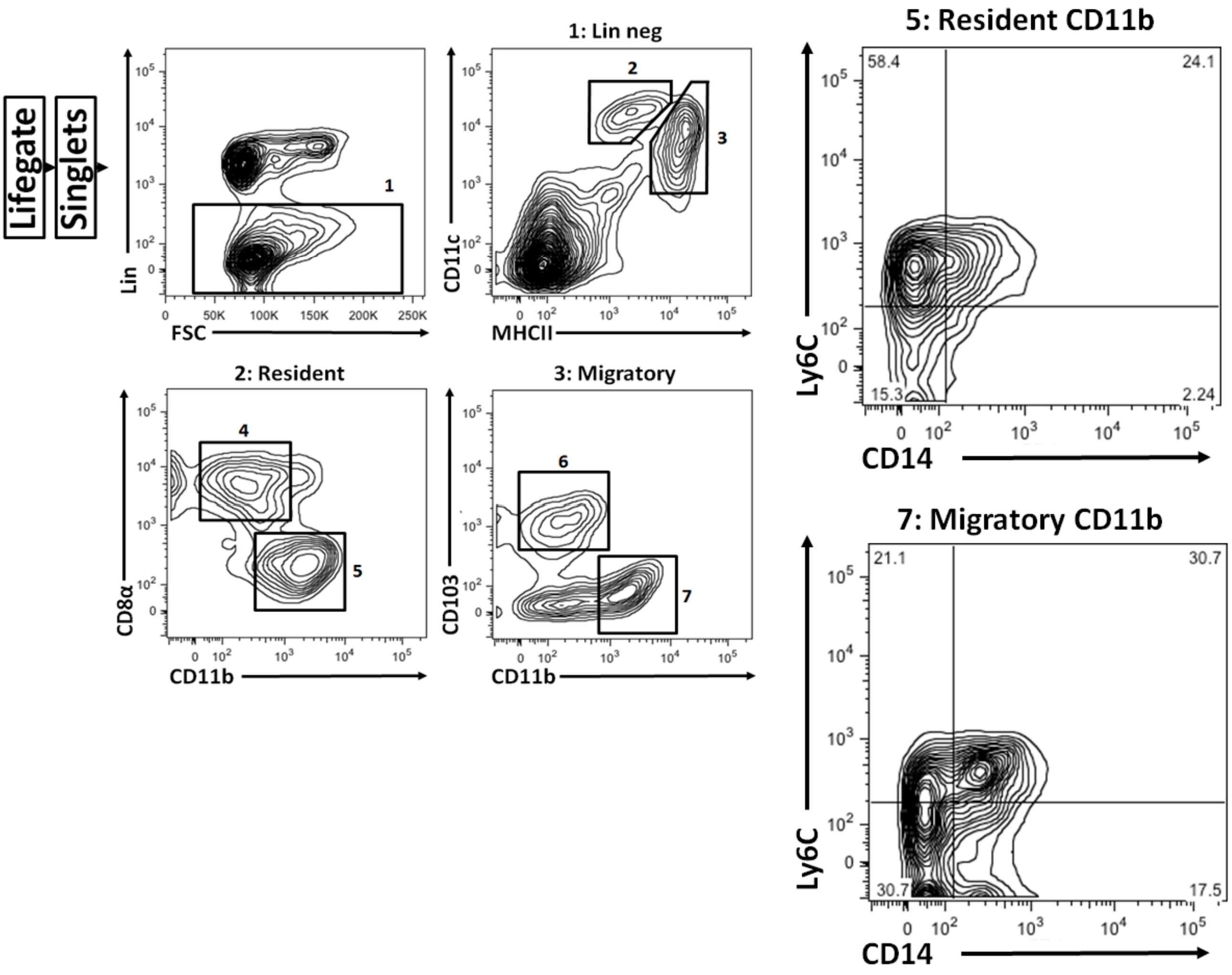

Supplementary figure 9. Monocytic markers on resident and migratory CD11b+ DCs from lymph node. Additional stainings were performed for the monocytic markers Ly6C and CD14 on the corresponding sorted cells from figure 5C.

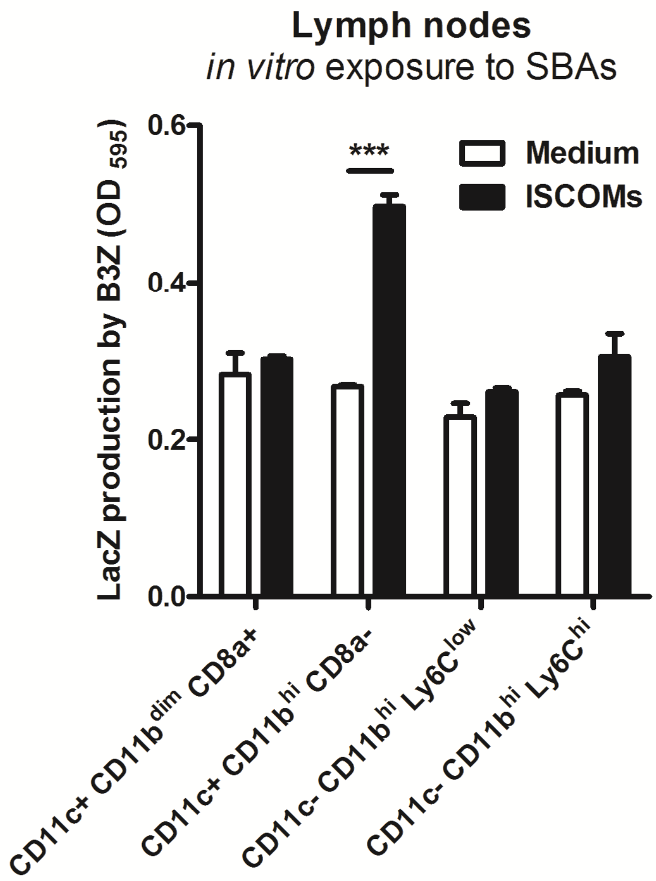

Supplementary figure 10. Following cryo-ablation, only the CD11b^{hi} DCs are able to cross-present OVA upon SBA exposure. Cross-presentation assay on *ex vivo* isolated DC subsets following *in situ* tumor ablation. Established B16F10 melanomas (5-8mm diameter) were treated with cryo ablation and 50 µg peritumorally injected CpG-ODN1668. 12 hours later draining lymph nodes were harvested and subjected to FACS sorting of four cell populations using the indicated markers. Different from the Flt3-L tumor assay in Fig. 5e, ablation with CpG does not induce a prominent pDC population (CD11c^{pos}CD11b^{neg}), but does create an influx of inflammatory monocytes (CD11c^{neg}CD11b^{hi}Ly6C^{hi}). The populations were exposed *in vitro* for 5 hrs to the indicated compounds (ISCOMs 800 ng/ml, OVA 300 µg/ml), after which cross-presentation was analyzed using the B3Z cell readout. Statistical analysis was done using two way ANOVA with post hoc Bonferroni tests.

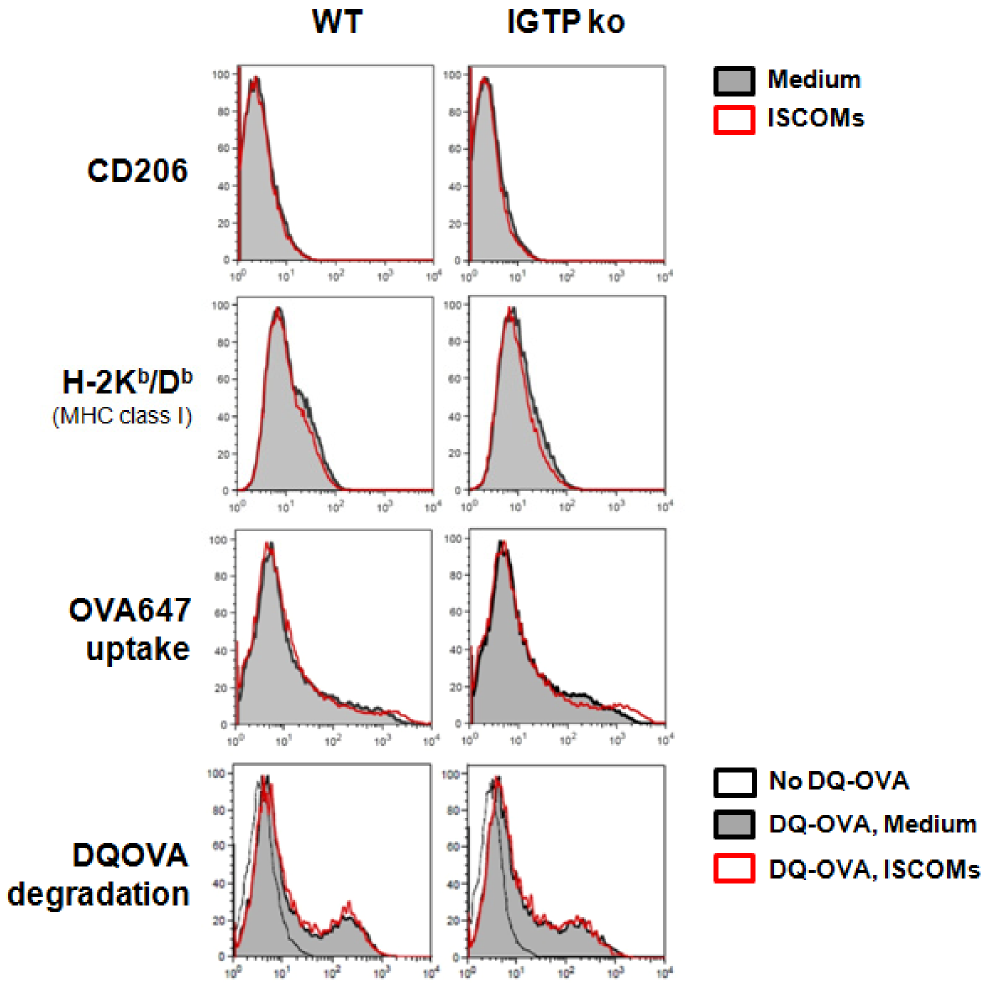

Supplementary figure 11. SBA-exposed IGTP -/- and +/+ BMDCs show equal levels of CD206, MHC-I (K^b/D^b), and takeup/degradation of OVA. FACS analysis of GM-CSF DCs generated from bone-marrow from IGTP -/- and +/+ mice. Cells were given medium, 0.25 µg/ml OVA coupled to the fluorophore Alexa647, or 1 µg/ml DQ-OVA, during the 5 hrs exposure time to medium or ISCOMs. DQ-OVA will start to fluoresce once degraded. Next, cells were processed for FACS stainings with anti CD206 (mannose receptor), or anti MHC-I antibodies. The filled black lines show the medium-treated cells, while the open red lines are the ISCOM treated samples.

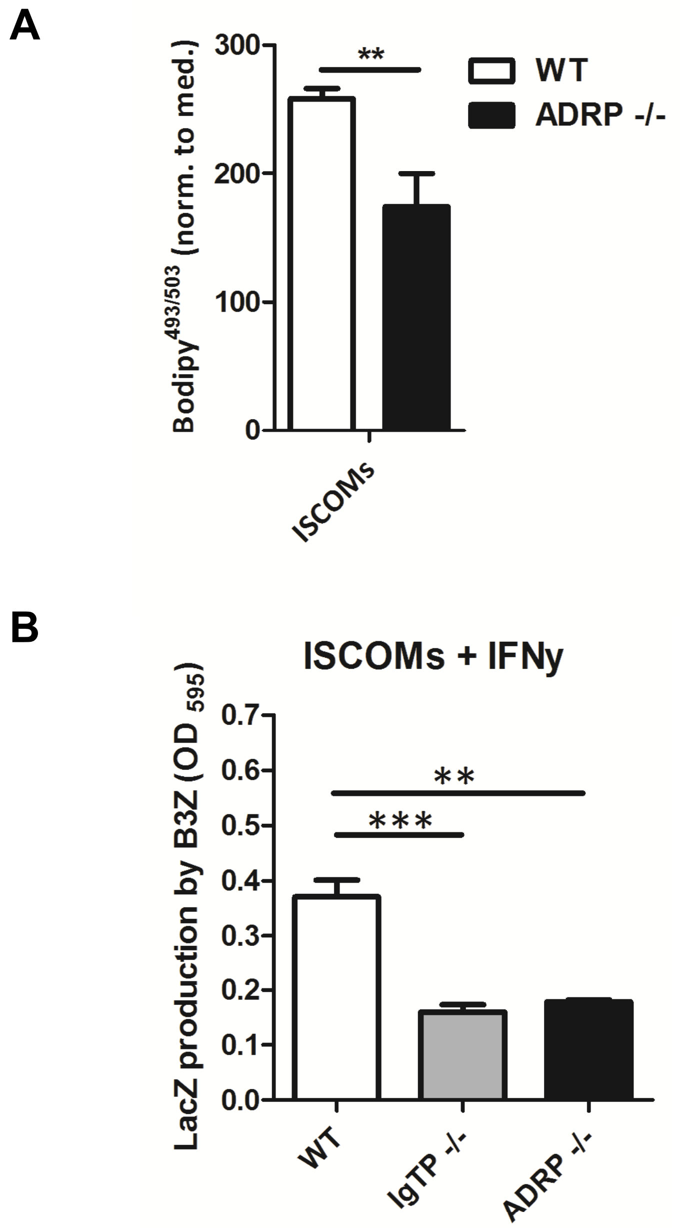

Supplementary figure 12. ADRP-/- BMDCs show lower levels of LBs and cross-presentation under stimulation with SBAs. (A) FACS analysis of GM-CSF DCs generated from bone-marrow from ADRP-/- and +/+ mice. Cells were stimulated for 5 hrs with ISCOMs, subsequently stained for LBs with the Bodipy dye, and analyzed by FACS. (B) ADRP or IGTP -/- cells were stimulated similar as above with ISCOMs, 250 ng/ml IFNγ, and OVA. Next, cross-presentation was determined using the B3Z assay. Statistical analyses were done using one way ANOVA with post hoc Bonferroni tests. Similar results were obtained in two independent experiments.

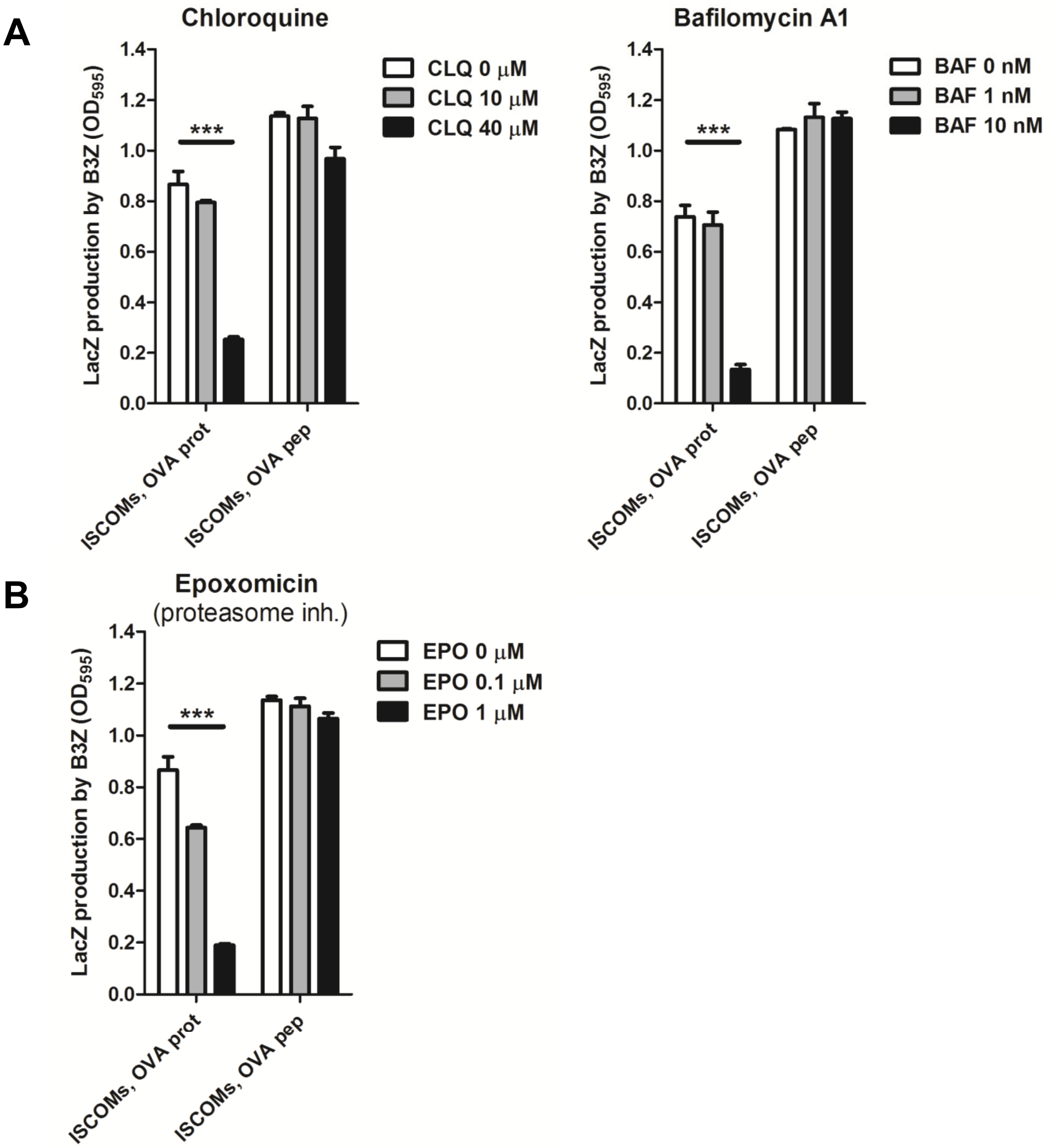

Supplementary figure 13. Endosomal acidification and the proteasome are needed for SBA-aided cross-presentation. *In vitro* cross-presentation assays after 5 hrs exposure of GM-CSF BMDCs to ISCOMs or the indicated concentrations of inhibitors of endosomal acidification ((A) Chloroquine and Bafilomycin A1) and the proteasome ((B) Epoxomicin). External peptide pulsing with OVA K^b peptide was used to control for viability and/or MHC-I levels. Statistical analysis was done using two way ANOVA with post hoc Bonferroni tests. Similar results were obtained in two independent experiments.

A

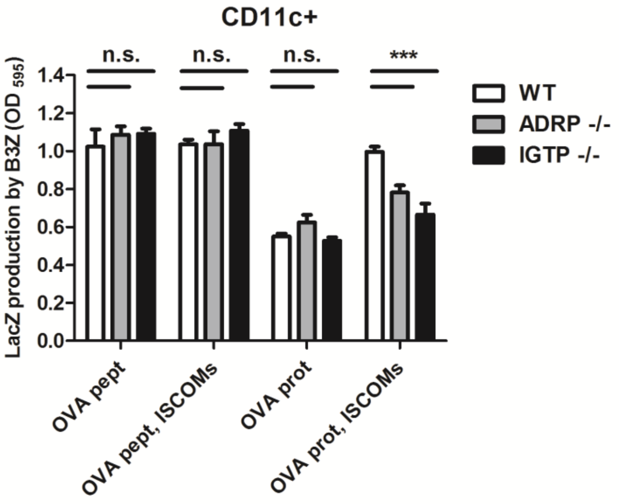

B

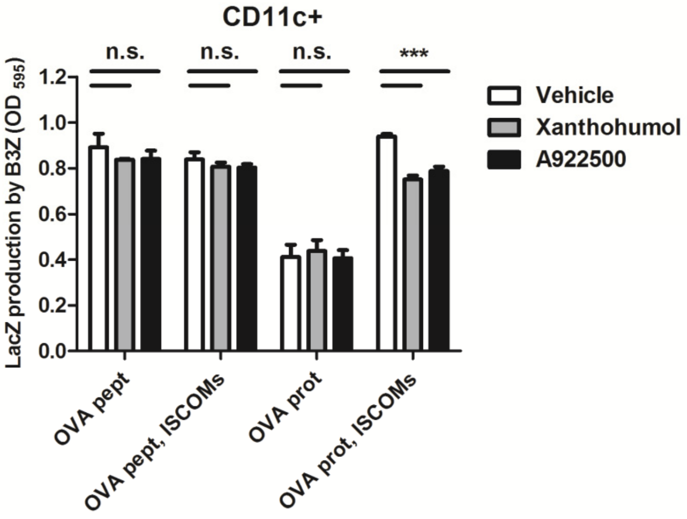

Supplementary figure 14. *In vivo* cross-presentation in ADRP or IGTP knockout mice, or after pharmacological LB inhibition. *In vitro* cross-presentation assays on *ex vivo* isolated lymph node DCs using B3Z cells as a readout. (A) Wild-type, IGTP-/-, or ADRP-/- knockout mice were injected with 300 µg endofree OVA protein or 40 µg OVA K^b peptide, with or without 30 µg ISCOMs. 12 hrs later, draining lymph nodes were harvested from which CD11c+ cells were isolated that entered the cross-presentations assays. B3Z cells were analyzed after 2 days. (B) Wild-type mice were injected s.c. on the femur with LB inhibitors (Xanthohumol: 500 µg, or A922500: 150 µg), or DMSO vehicle control. Four hrs later, identical injections were given, this time combined with 300 µg endofree OVA protein or 40 µg OVA K^b peptide, with or without 30 µg ISCOMs. After 12 hrs, CD11c+ cells from lymph nodes were isolated and analyzed similar as under (A). Statistical analysis was done using two way ANOVA with post hoc Bonferroni tests.

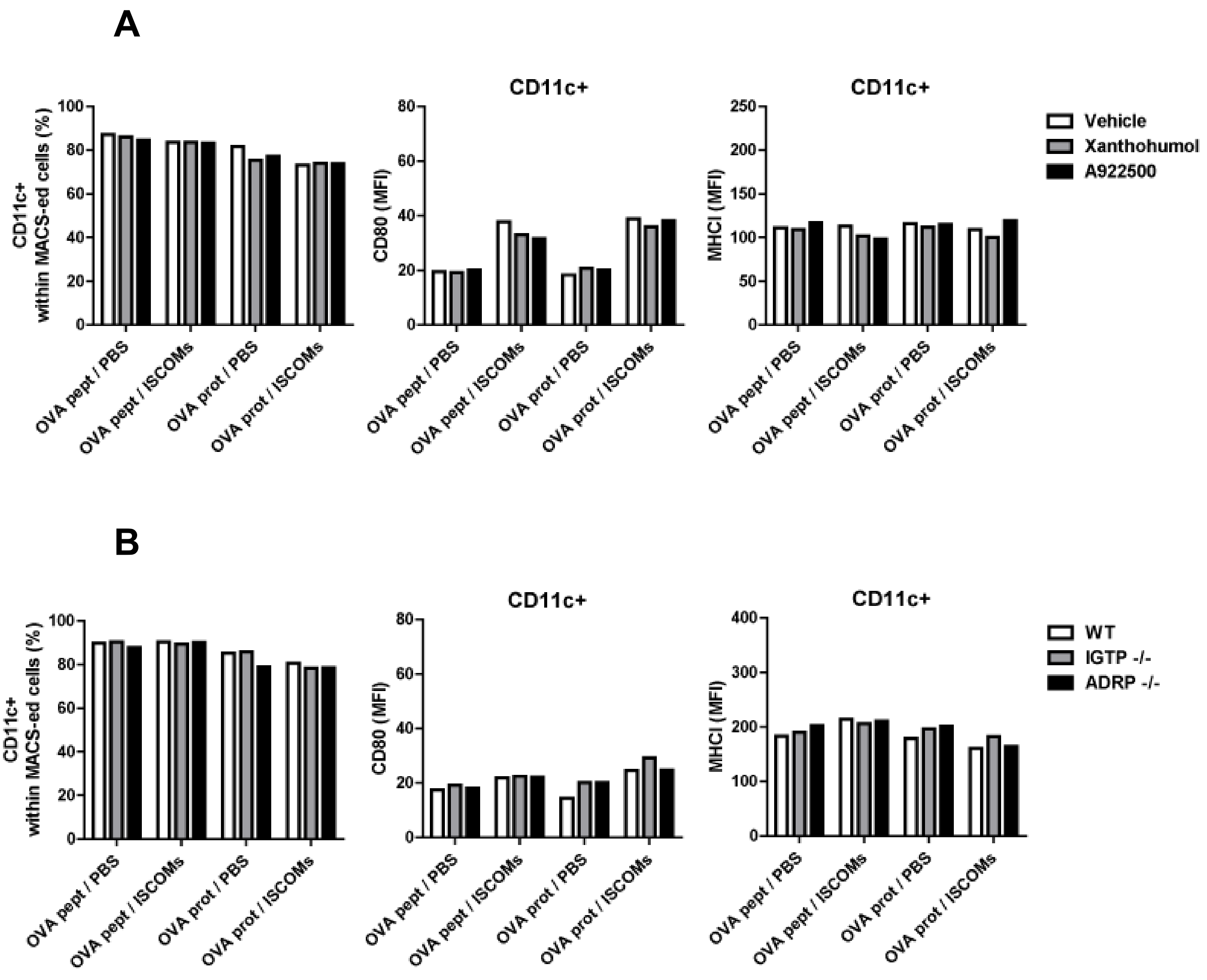

Supplementary figure 15. CD11c percentage, or CD80/MHC-I expression of MACS sorted DCs is unchanged in LB inhibitor-treated mice or IGTP/ADRP knockouts. FACS analysis of cells as isolated under figures 6J and S14. (A) Mice were injected s.c. on the femur with 500 µg Xanthohumol, 150 µg A922500, or DMSO vehicle control. Four hrs later, identical injections were given, this time combined with 300 µg endofree OVA protein or 40 µg OVA K^b peptide, with or without 30 µg ISCOMs. 12 hrs later, draining lymph nodes were harvested from which CD11c+ cells were isolated by magnetic bead sorting. (B) Wild-type, IGTP-/-, or ADRP-/- knockout mice were injected with 300 µg endofree OVA protein or 40 µg OVA K^b peptide, with or without 30 µg ISCOMs. 12 hrs later, draining lymph nodes were harvested from which CD11c+ cells were isolated. Data represent single values. Similar results were obtained in two independent experiments.

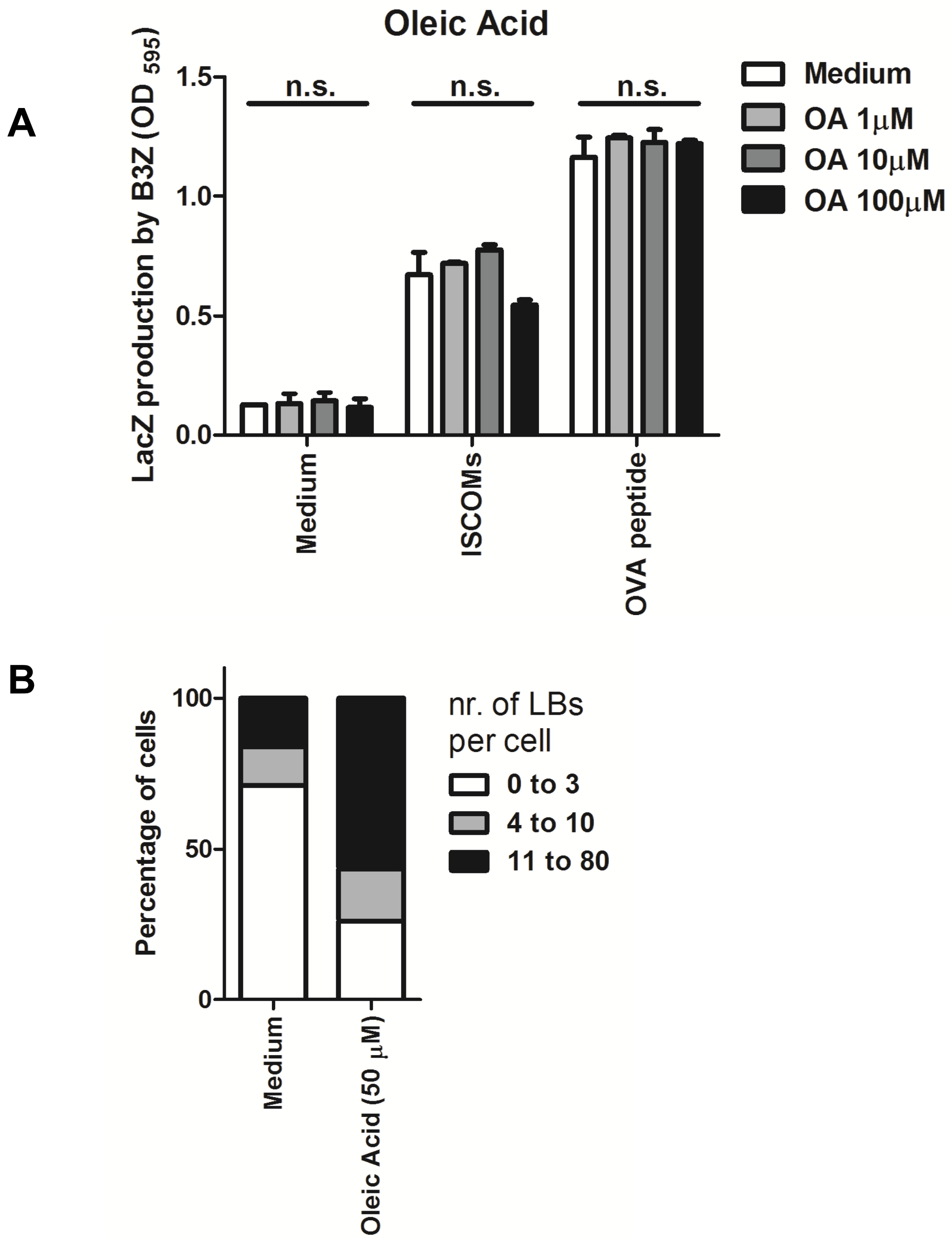

Supplementary figure 16. Induction of lipid bodies with oleic acid does not induce cross-presentation, and has minor influence on SBA-induced cross-presentation. *In vitro* cross-presentation assay (A) and quantification of LBs (B) in GM-CSF BMDCs following 5 hrs exposure to ISCOMs or 50 µM oleic acid. Similar data were obtained in two independent experiments. Statistical analysis was done using two way ANOVA with post hoc Bonferroni tests.

Supplementary References

1: *Mayer CT, et al. Selective and efficient generation of functional Batf3-dependent CD103+ dendritic cells from mouse bone marrow. Blood 124, 3081-3091 (2014).*
